# Identification of bioactive compounds of *Bacillus velezensis* HNA3 that contribute to its dual effects as plant growth promoter and biocontrol against post-harvested fungi

**DOI:** 10.1128/spectrum.00519-23

**Published:** 2023-10-09

**Authors:** Doaa S. Zaid, Wenya Li, Siyu Yang, Youguo Li

**Affiliations:** 1 State Key Laboratory of Agricultural Microbiology, Huazhong Agricultural University, Wuhan, China; 2 Desert Research Center, Ain Shams, Egypt; Environmental Microbiology, Institute of Ecology INECOL, Pátzcuaro, Michoacán, Mexico

**Keywords:** *Bacillus velezensis* HNA3, volatile and semi-volatile organic compounds (VOCs), post-harvested phytopathogen, fatty acid methyl ester (FAME), gas chromatography-mass spectrometry (GC-MS), gene expression (RT-qPCR)

## Abstract

*Bacillus velezensis* HNA3 displayed dual bioactivities as a plant growth promoter and a biocontrol agent against five isolated post-harvest phytopathogens: *Alternaria alternata*, *Cladosporium cladosporioides*, *Penicillium expansum*, *Monilinia fructicola*, and *Fusarium oxysporum*. Moreover, the volatile organic compounds (VOCs) emitted by HNA3 exhibited remarkable efficacy, achieving 100% fungal inhibition against *Alternaria alternata* and *Cladosporium cladosporioides*. This is the initial study to demonstrate the HNA3-emitted VOCs effect on enhancing soybean seedling growth and seed germination by breaking seed dormancy and improving root construction. Semi-VOCs and emitted VOCs were identified by two methods of extraction coupled with gas chromatography-mass spectrometry. A total of 14 main VOCs were detected; phenol,2,4-bis(1,1-dimethylethyl) and 1,2-benzenedicarboxylic acid are the most abundant semi-VOCs, both are known to have anti-fungal and plant growth-promoting properties. 9-Octadecenoic acid (z)-, methyl ester is the most common volatile compound emitted by HNA3 followed by hexadecanoic acid, methyl ester and heptadecanoic acid, methyl ester. It is noteworthy that heptadecanoic acid, methyl ester has not been previously detected in *Bacillus* spp. Pure 9-octadecenoic acid (z)-, methyl ester, as well as heptadecanoic acid, methyl ester, exhibited inhibitory effects on the fungal growth of all isolated phytopathogens. Interestingly, when combined, these compounds synergistically eradicated fungal growth. Indole acetic acid and serotonin, both classified as plant growth-promoting indoles, have been identified within HNA3. Moreover, during the phytopathogen inhibition stage, HNA3 demonstrated upregulation in gene expression associated with non-VOCs, including bacillibactin, bacillomycin, bacilysin, and surfactin. This study publicized an extensive variety of bioactive compounds produced by HNA3 liable for its bioactivities.

**IMPORTANCE:**

The current study is an extension to our previous work on the plant growth-promoting rhizobacteria (PGPR) *Bacillus velezensis* HNA3 strain, which comes to confirm and reveals the huge stock of active secondary metabolites produced by HNA3. HNA3-emitted volatile organic compounds (VOCs) have demonstrated the capacity to impede the growth of phytopathogens affecting some fruits and vegetables, even in the absence of direct contact. Additionally, these volatiles enhanced soybean seed germination by breaking seed dormancy and inducing root system development. Furthermore, they promoted seedling growth, giving it prominence in soybean cultivation. The relevance of active volatiles derives from the fact that they can be developed as natural-safe biocontrol agents and plant promoters. This research validates the remarkable bioactivities exhibited by the *Bacillus velezensis* HNA3 and their potential applications in agriculture as an inoculant, encompassing biocontrol, plant growth promotion, and seed germination activities, thereby offering a safer alternative to hazardous chemicals.

## INTRODUCTION

Agriculture has faced various issues in recent years, including decreases in yield output, phytopathogen resistance, soil fertility depletion, and global climate change (1). These issues demand researchers and studies to develop acceptable solutions to ensure the countries’ food security (2). The majority of existing treatments for agriculture issues have negative consequences on both health and the environment (3). As per sustainable agriculture perspectives, controlling diseases and increasing crop yield and quality are the most significant challenges nowadays, so it has become an urgent necessity to employ methods from natural sources to avoid polluting the environment and keep the general health of animals and humans (3).

Fruits and vegetables represent vital dietary sources abundant in essential nutrients. They serve both as nourishment and, in certain cases, as medicinal agents. These commodities are easily obtainable and ingestible. Furthermore, they can be consumed in their natural raw state, obviating the requirement for cooking (4). Nevertheless, their vulnerability to fungal infections post-harvest is notable, contributing to post-harvest losses exceeding one-third of the overall yield in fruit crops (5). Among the most widely recognized fungi that attack fruits and vegetables during and after harvest are *Penicillium*, *Alternaria*, *Botrytis*, *Geotrichum*, *Fusarium*, *Monilinia*, *Cladosporium*, and others (6).

Post-harvest phytopathogens are necrotrophic fungi that not only inflict damage upon plant cells but also exploit their contents for proliferation, giving rise to potentially perilous secondary compounds recognized as mycotoxins (7). If they are not effectively handled, they spread rapidly causing full crop destruction. They are regarded as the primary cause of agricultural losses, as well as a serious threat to human health because of the fruits and vegetables' contamination with fungal mycotoxins (8).

*Alternaria alternata* stands as one of the prevalent fungi that post-harvest infect vegetables and fruits. This pathogen inflicts damage across diverse regions worldwide and induces food poisoning through the generation of multiple carcinogenic mycotoxins (9). Furthermore, blue mold disease presents as another potent post-harvest ailment affecting apple fruits. This affliction, attributed to *Penicillium expansum*, gives rise to substantial economic losses during the storage of apples and significantly impacts fruit quality (10). Both Fusarium dry rot and Fusarium wilt are serious potato diseases caused by *Fusarium oxysporum* (11). Because of post-harvest losses, these diseases yearly lower potato production by 25%–60%. In addition, certain *Fusarium* spp. that cause dry rot also produce mycotoxins that can poison people and farm animals (12). *Cladosporium cladosporioides* emerges as a post-harvest pathogen that triggers infections in numerous fruits and vegetables, encompassing tomatoes, grapes, sweet peppers, and others. This pathogen inflicts significant harm to host plants during storage, and currently, there is a lack of effective fungicides for its control. As a result, there exists a pressing need to develop a safe biocontrol agent to combat this issue (13). Brown rot disease occurs during the post-harvest period of stone fruit due to the infection with *Monilinia* spp. forms an economic challenge because of the severe losses it causes to the stone fruit crop (14).

Rhizobacteria, naturally inhabiting plant roots, confer numerous advantages to plants, encompassing disease prevention and facilitation of plant growth (15). Several PGPRs genera exhibit unique attributes and diverse metabolic pathways. However, *Bacillus* spp. hold prominence in applied techniques, such as the formulation of bio-fertilizers and bio-fungicides, owing to their multifaceted biological activities, synthesis of potent metabolites, and proficiency in spore formation, which enhances cell viability in commercially manufactured products (16 – 18). *Bacillus velezensis* PGPR has recently garnered attention due to its unique genetic characteristics within the *Bacillus* genus. This bacterium allocates the majority of its gene clusters toward the synthesis of diverse bioactive compounds (17). This genetic propensity underpins its multi-functional characteristics, granting it a wide array of beneficial functions (19). *Bacillus licheniformis* K11 inhibits the growth of phytopathogen *Phytophthora* sp., enhances the growth of pepper plants and produces indole acetic acid (IAA) and lytic enzymes (20). *Bacillus velezensis* Ag75 multi-functional agent promotes maize and soybean growth, solubilizes phosphate, and suppresses the growth of pathogenic fungi including *Rhizoctonia solani*, *Fusarium solani*, and *Macrophomina phaseolina* (19). *Bacillus* sp. GH1-13 shows multi-functional activities, including the biocontrol effect against pathogenic fungi associated with rice as well as promoting rice growth and producing IAA (21). Volatile organic compounds (VOCs) produced by *Bacillus amyloliquefaciens* L3 showed anti-fungal against post-harvested Fusarium wilt and promoted the growth of *Arabidopsis thaliana* (22). Additionally, *Bacillus* sp. BCT9 produces VOCs that promote plant growth and enhance seed germination of the *Lactuca sativa* plant (23). *Bacillus mojavensis* I4 produces VOCs that have an inhibition activity toward pathogenic *Fusarium* sp. and *Rhizoctonia* sp. besides their growth promotion effect on Arabidopsis plants (24).

The study makes significant contributions to the identification of the secondary metabolite fingerprint of the *Bacillus velezensis* HNA3 strain. Our investigation delved deeply into the biological activities exhibited by both volatile and non-volatile compounds. Specifically, we explored the anti-fungal properties, enhancement of seed germination, and stimulation of plant growth exerted by the PGPR *Bacillus velezensis* HNA3.

### The objectives of this study

The objective of this study is to investigate the anti-fungal activity of VOCs and non-VOCs produced by the HNA3 strain against the five isolated phytopathogenic fungi. In addition, we will study the plant growth-promoting effect of the HNA3 strain by adding HNA3 broth culture to the soybean plant (in pot experiment) and by exposing soybean seeds and seedlings to volatiles emitted by HNA3 (plate assay and pot experiment). Qualitative and quantitative colorimetric estimation of indole acetic acid generated by HNA3 will be conducted. We will use gas chromatography-mass spectrometry (GC-MS) to identify both semi-VOCs and emitted VOCs by two methods of extraction, ethyl acetate solvent extraction (SE) and headspace solid-phase microextraction (HS-SPME). Additionally, we will assess the anti-fungal effect of some individual pure VOCs and their mixture. Finally, in order to have a complete view of the metabolic profile of HNA3, we will investigate gene expression regulation for eight genes predicated to produce non-volatile lipopeptides and polypeptides secondary metabolites using reverse transcription-quantitative polymerase chain reaction (RT-qPCR).

## RESULTS

### Isolation and molecular identification of five post-harvested fungi

In this study, five different post-harvested fungal strains were isolated from some rot fruits and vegetables and went through several purification steps then identified based on their colony morphological characteristic and specific features of the developed conidia/spores. *Alternaria* sp. and *Cladosporium* sp. were isolated from tomatoes, while *Penicillium* sp., *Monilinia* sp., and *Fusarium* sp. were isolated from apples, peaches, and potatoes, respectively. The morphological and microscopic identification of the isolated fungal strains is illustrated in Fig. S1. Results of basic local alignment search tool (BLAST) analysis of internal transcribed spacer (ITS) or 18S rRNA sequence of the isolated pathogenic fungal strains showed 98%–99% identity with closely related fungal strains in the national center for biotechnology information (NCBI) database. The outcomes of this sequence analysis were consistent with those of the morphological identification and molecularly identified as *Alternaria alternata*, *Penicillium expansum*, *Monilinia fructicola*, *Fusarium oxysporum*, and *Cladosporium cladosporioides*. The results of genetic analysis and accession number for each isolate are illustrated in Table 1, while the phylogenetic trees are shown in Fig. S2 to S4.

**TABLE 1:** Results of sequence analysis of ITS or 18S ribosomal RNA (18S rRNA) genetic region for isolated post-harvested pathogenic fungal strains

Species identified	Primer used	Sequence length	Length of overlap sequence	Similarity, %	Closest accession number	GenBank accession number
*Alternaria alternata*	ITS1, ITS2	560	549	98	KY676196.1	OQ216618
*Penicillium expansum*	ITS1, ITS2	602	595	99	MT872092.1	OQ201736
*Monilinia fructicola*	ITS1, ITS2	472	459	98	LT615175.1	OQ216738
*Fusarium oxysporum*	ITS1, ITS2	460	456	99	ON945580.1	OQ216619
*Cladosporium cladosporioides*	NS1, NS25	1656	1653	99	MG769026.1	OQ201835

### Anti-fungal activity of HNA3

**Fig 1:**
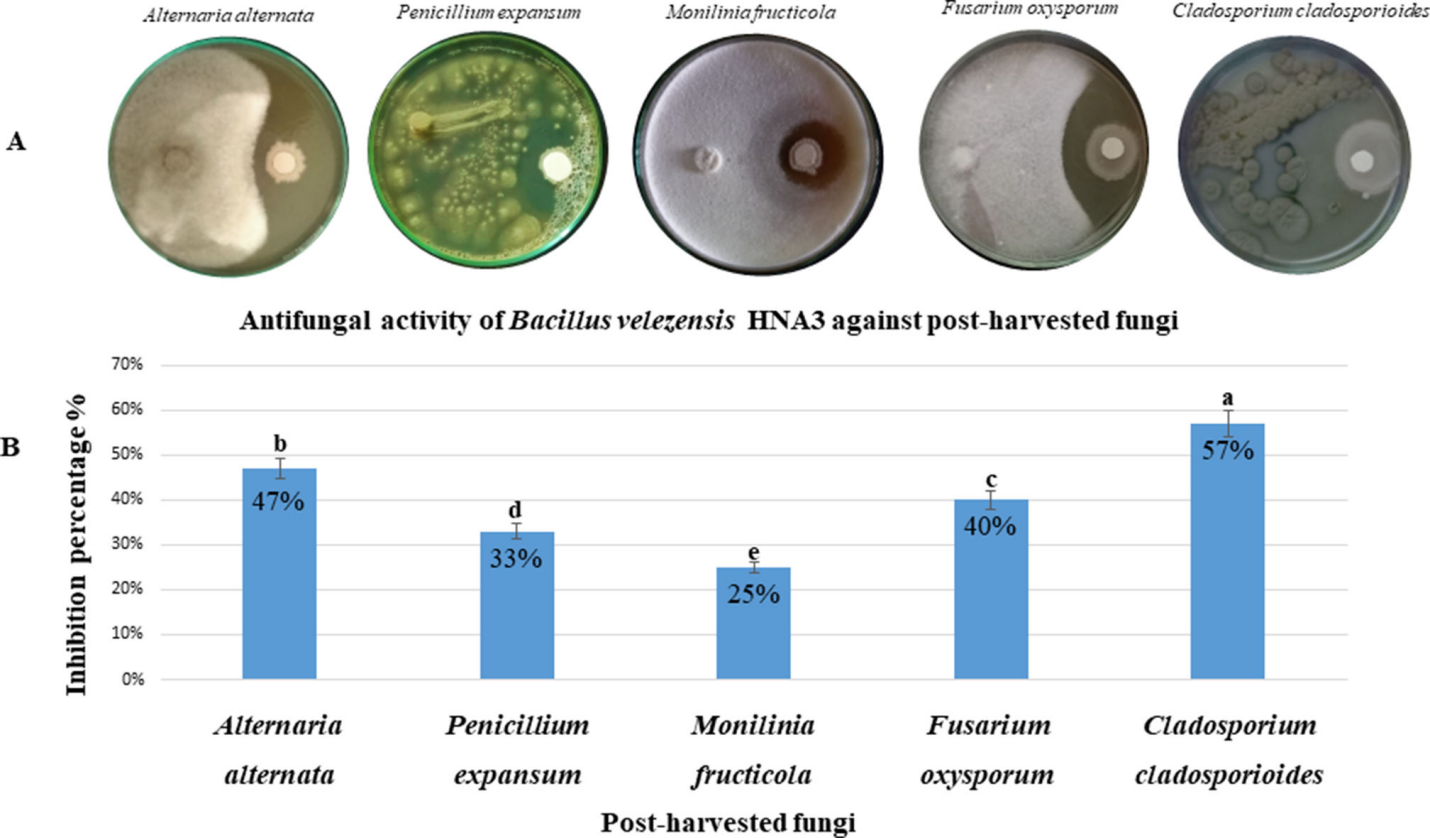
The antagonist activity of HNA3 against isolated post-harvest pathogenic fungi. (A) HNA3 exhibits inhibition of the growth of isolated post-harvest pathogenic fungi on potato dextrose agar (PDA) plates at 25°C. (B) The percentage of inhibition is graphically represented, with values presented as the mean ± standard deviation (SD) from three replicates (*n* = 3). Letters “a, b, c, d, and e” denote significant differences (*P* < 0.05) as determined by Tukey’s honestly significant difference (HSD) test.

Active metabolites secreted by the HNA3 strain could inhibit the mycelial growth of the isolated post-harvested fungi with different inhibition percentages ranging from 57% to 25% (*P* < 0.05). HNA3 strain displayed significant growth inhibition of *Cladosporium cladosporioides* and *Alternaria alternata* with percentages of 57% ± 0.7% and 47% ± 0.6%, respectively, while it had moderate growth inhibition against *Fusarium oxysporum* with an inhibition percentage of 40% ± 0.6%, *Penicillium expansum* with inhibition percentage of 33% ± 0.9%, and *Monilinia fructicola* with inhibition percentage of 25% ± 0.5%. HNA3 inhibition rate against post-harvested fungi is illustrated in Fig. 1.

### Anti-fungal activity of emitted volatiles produced by HNA3

**Fig 2:**
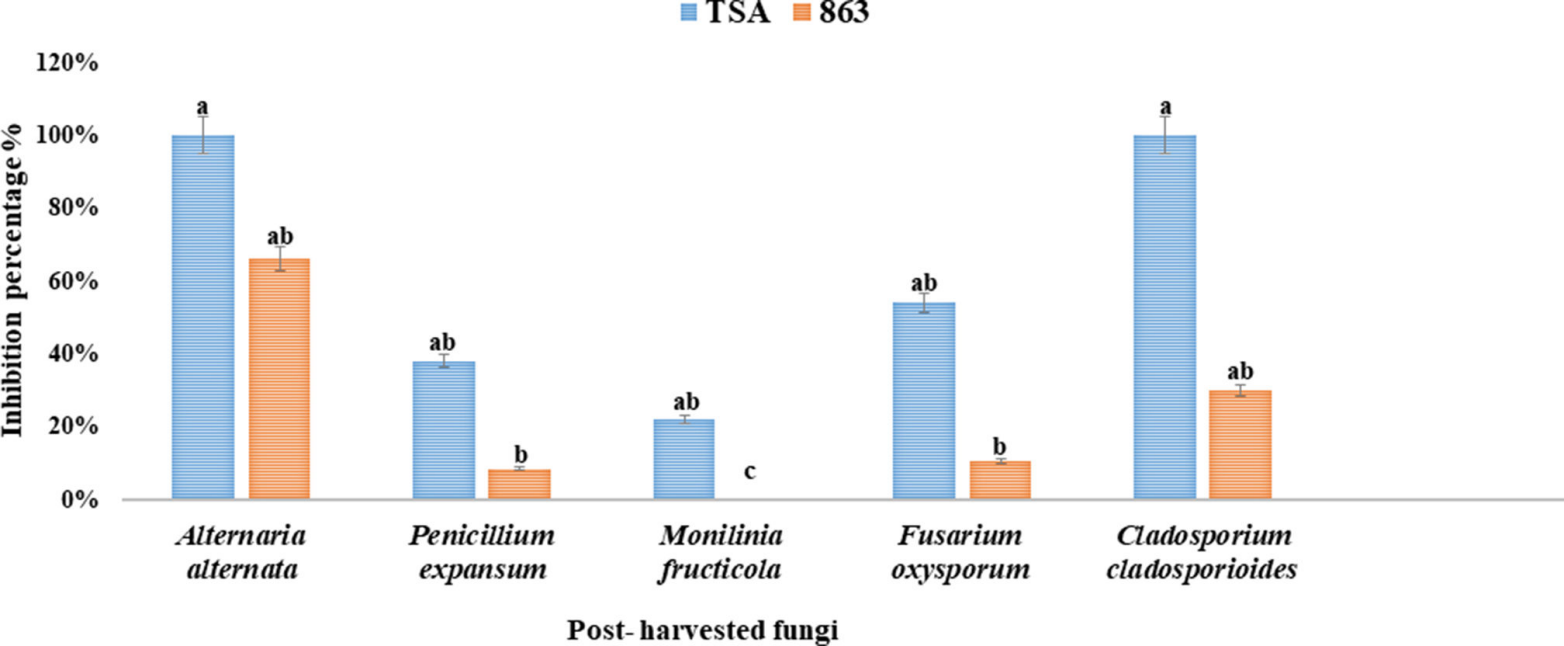
The inhibition percentage of post-harvested fungal isolates following treatment with VOCs emitted by HNA3 in two different types of media. All data are presented as the mean ± SD with *n* = 3 replicates. Letters “a, ab, b, and c“ indicate significant differences in the inhibition percentage between the two treatments, determined through one-way analysis of variance (ANOVA) and Tukey’s HSD test (*P <* 0.05).

VOCs emitted by the HNA3 strain have a biocontrol activity against the isolated post-harvested fungi (Fig. S5). When employing tryptone soy agar (TSA) media for cultivating HNA3, it was observed that volatiles produced the most potent inhibitory impact on fungal growth. The inhibition percentage ranged from 100% ± 0.9% and 100% ± 0.8% (*P* < 0.05) growth inhibition of *Alternaria alternata* and *Cladosporium cladosporioides* to 54% ± 1%, 38% ± 0.9%, and 22% ± 1% (*P* < 0.05) growth inhibition of *Fusarium oxysporum*, *Penicillium expansum*, and *Monilinia fructicola*, respectively. Volatiles showed less inhibitory effect using 863A media for the growth of HNA3 ranging from 66% ± 0.9%, 30% ± 0.9%, 10.5% ± 1%, 8.5% ± 0.9%, to 0% ± 1% (*P* < 0.05) growth inhibition of *Alternaria alternata*, *Cladosporium cladosporioides*, *Fusarium oxysporum*, *Penicillium expansum*, and *Monilinia fructicola*, respectively (Fig. 2).

### Plant growth-promoting activity of HNA3

**Fig 3:**
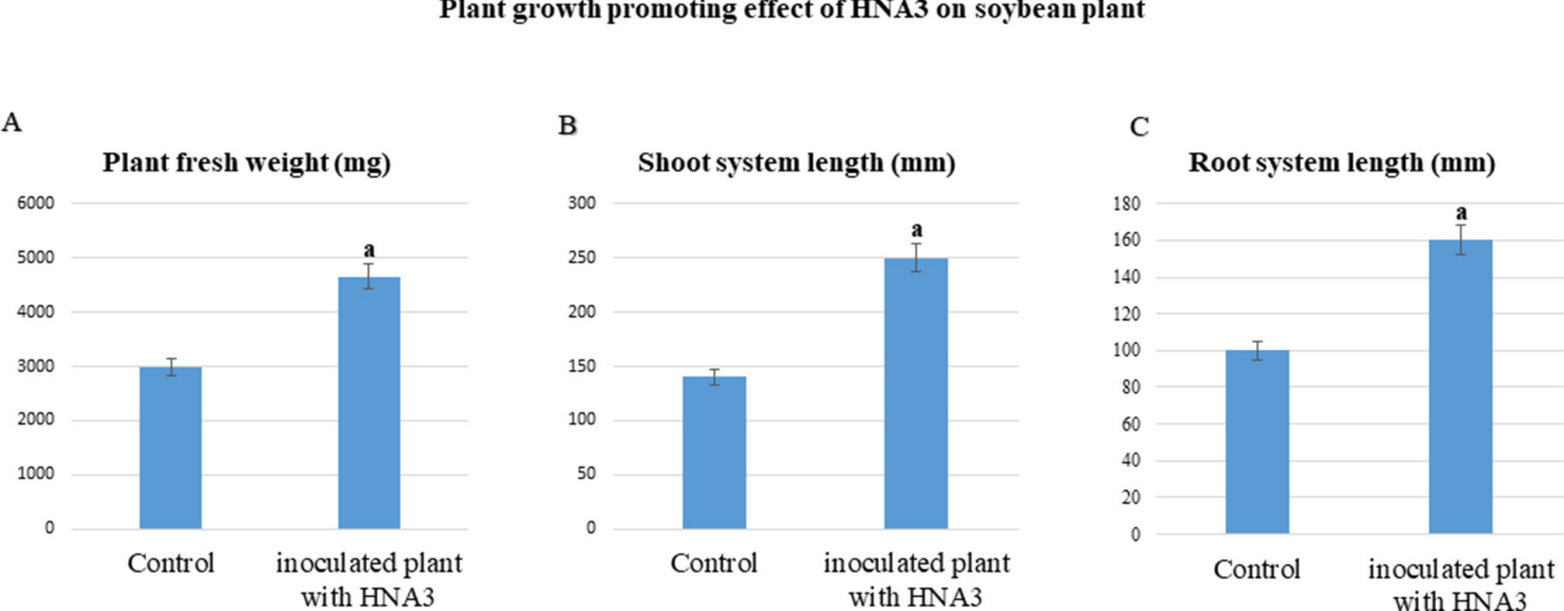
The plant growth-promoting impact of HNA3 on soybean. (A) The fresh weight of soybean plants, (B) the shoot length, and (C) the length of the root system are shown. The bars in the graph indicate the mean ± SD values from six replicates (*n* = 6). The letter “a” signifies significant differences between conditions, as determined by the Tukey’s HSD test (*P* < 0.05).

An in-pot experiment was conducted to assess the plant growth-promoting activity of the bioactive compounds excreted by HNA3 on soybean plant growth. Upon a general observation of the phenotypes resulting from the two treatments (plants inoculated with HNA3 and non-inoculated control plants), a notable increase in plant biomass, and the emergence of multiple branches within the root system were observed in the inoculated plants compared to the control plants. According to the measurements, inoculated plants measured 250 ± 4 mm and 160 ± 3 mm for shoot and root length compared with control plants that measured 140 ± 2 mm and 100 ± 3 mm. The fresh weight of the inoculated plants is nearly double the weight of the control plants with measurements of 4,660 ± 6 mg for inoculated plants and 2,980 ± 5 mg for the control plants (*P* < 0.05) (Fig. 3).

### Seed germination enhancement and seedling growth promotion of emitted VOCs produced by HNA3

**Fig 4:**
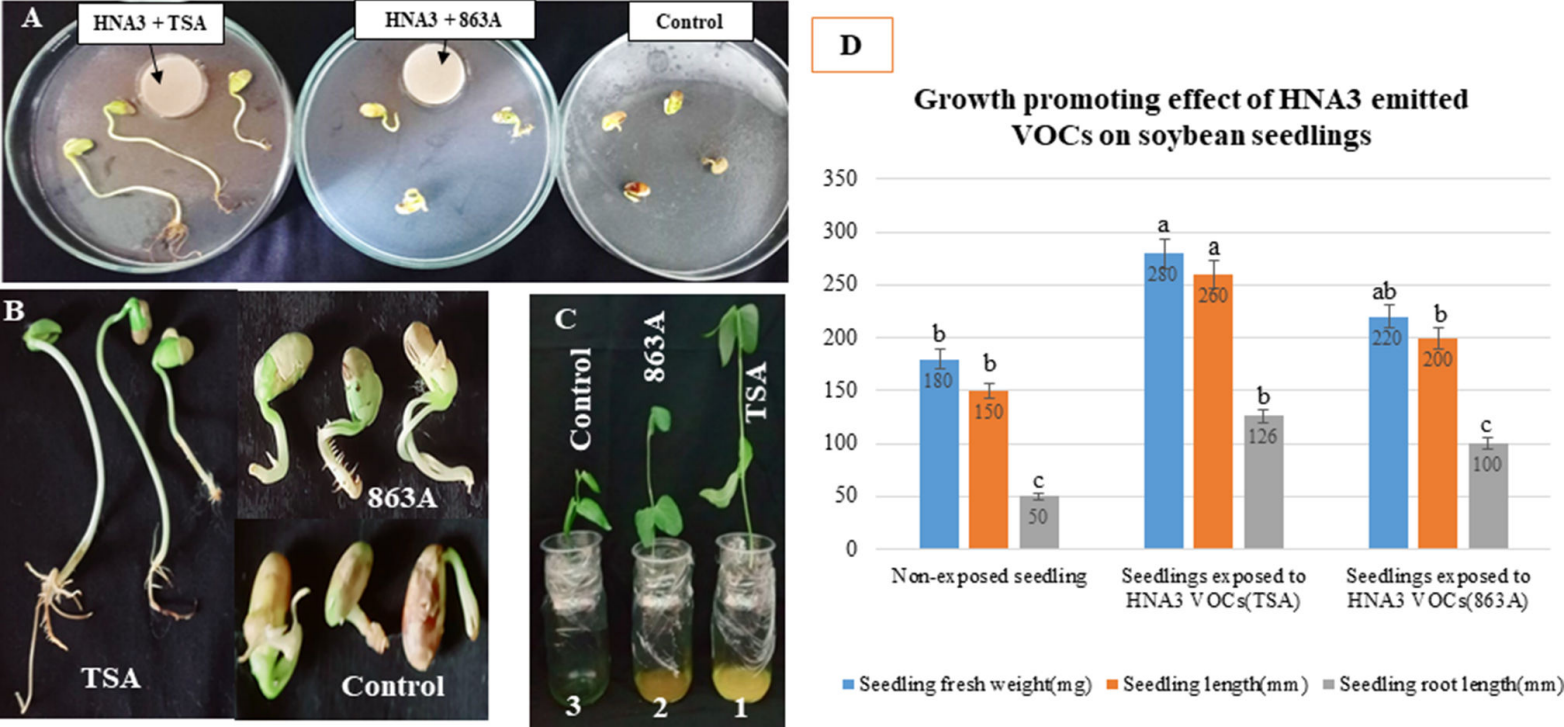
The impact of VOCs emitted by HNA3 on soybean seed germination and seedling growth. (A) The promoting effect of HNA3-emitted VOCs on the germination of soybean seeds using two different growth media for HNA3 (TSA and 863A). This germination was assessed after 15 days of incubation in a light chamber under conditions of 16 hours of light and 8 hours of darkness at 30°C ± 2°C. Seeds in the control plate were not exposed to any treatment. (B) The appearance of germinated seeds after exposure to HNA3-emitted VOCs using both TSA and 863A media. (C) The growth-promoting effect of HNA3-emitted VOCs on soybean seedlings after 15 days of growth in a light chamber under 16 hours of light and 8 hours of darkness at 30°C ± 2°C. Three conditions are shown as follows: (1) seedlings with the best growth using TSA as the growth medium for HNA3, (2) seedlings with moderate growth using 863A as the growth medium for HNA3, and (3) non-exposed seedlings (control). (D) Various growth parameters after exposing seedlings to HNA3-emitted VOCs. All data are presented as the mean ± SD from six replicates (*n* = 6). Letters “a, ab, b, and c” indicate significant differences, determined through one-way ANOVA and Tukey’s HSD test (*P* < 0.05).

In plate assay, soybean seeds showed enhanced germination after being exposed to VOCs emitted from HNA3 using both TSA and 863A as growth media than non-exposed seeds (control) (Fig. 4A). Overall, there was a notable increase in the germination percentage of the exposed seeds compared to the non-exposed ones. Specifically, the germination percentage for the non-exposed seeds (control) stood at 33% ± 2%, whereas it reached 85% ± 1% and 66% ± 2% (*P* < 0.05) for the exposed seeds when utilizing TSA and 863A media for the growth of HNA3, respectively. However, seeds that have developed the best germination, and significant enhancement in primary and bilateral roots growth were observed using TSA. Primary root growth of the exposed seeds increased by 10.6-fold and the number of the bilateral roots by 12.3-fold compared with the non-exposed seeds as illustrated in Fig. 4B. We also noticed that, under the same conditions, the exposed seeds germinated within 4 days, unlike the non-exposed seeds that did not have any change. This suggests that VOCs produced by HNA3 broke the dormancy of the soybean seeds and improved their germination.

In the pot assay, following exposure of soybean seedlings to VOCs released through five holes at the base of the planting pot, a notable increase was observed in the fresh weight of the seedlings, their length, and the length of their root systems, when compared to the seedlings that were not exposed (control). Given that HNA3 was cultivated in two distinct media (TSA and 863A), the VOCs emitted from HNA3 subsequent to growth on TSA media exhibited more pronounced growth-promoting effects on soybean seedlings (Fig. 4C). The exposed seedlings' fresh weight measured 280 ± 2 mg when exposed to VOCs from TSA-grown HNA3 and 220 ± 1 mg when exposed to VOCs from 863A-grown HNA3. In comparison, the non-exposed seedlings weighed 180 ± 1 mg. The seedlings' length reached 260 ± 1 mm for those exposed to VOCs from TSA-grown HNA3 and 200 ± 3 mm for those exposed to VOCs from 863A-grown HNA3, whereas the non-exposed seedlings measured 150 ± 3 mm. Notably, the root length of the exposed seedlings was twice that of the non-exposed seedlings in both TSA and 863A media (*P* < 0.05) as shown in Fig. 4D.

### IAA production

**Fig 5:**
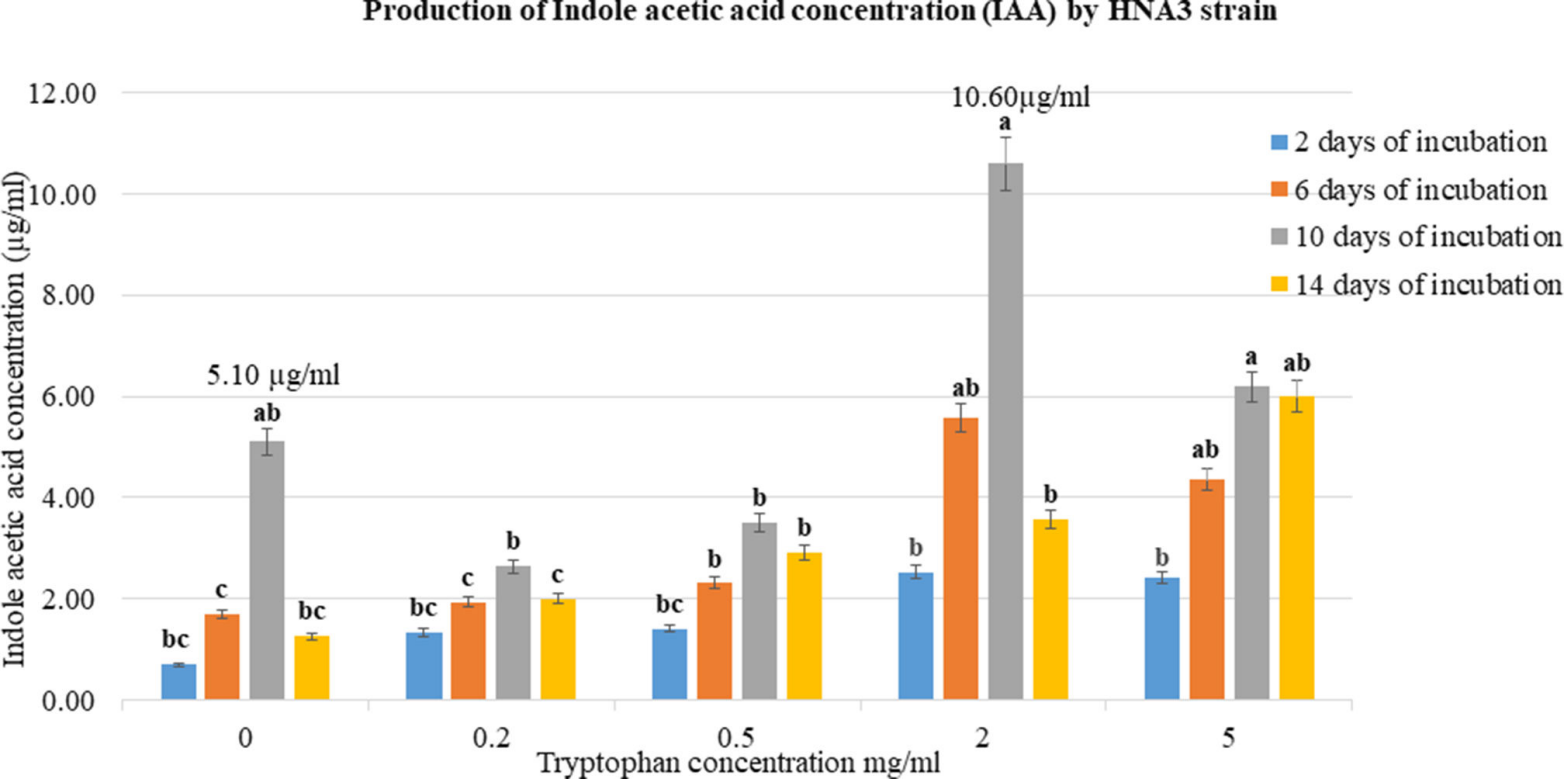
The production of IAA by the HNA3 strain. The highest amount of IAA (10.60 µg/mL) was produced by HNA3 after 10 days of incubation in the dark at 37°C, in the presence of 2 mg/mL tryptophan concentration. Additionally, it produced 5.10 µg/mL of IAA in the absence of tryptophan. All data are presented as the mean ± SD from 12 replicates (*n* = 12). Letters “a, ab, b, c, and bc“ denote significant differences in indole acetic acid concentrations, as determined through one-way ANOVA and Tukey’s HSD test (*P* < 0.05).

The colorimetric assay has been employed to measure IAA concentration in HNA3 broth. HNA3 strain has the ability to produce phytohormone IAA after 10 days of incubation period in the dark at 37°C. HNA3 produced IAA in the absence and presence of tryptophan (precursor of IAA). HNA3 produced 10.60 ± 0.03 µg/mL (*P* < 0.05) IAA after 10 days of incubation in the existence of 2 mg/mL tryptophan concentration. Also, produced 5.10 ± 0.04 µg/mL (*P* < 0.05) of IAA in the absence of tryptophan (Fig. 5). Concentrations of IAA have been calculated from the standard curve (Fig. S6).

### Identification of semi-volatiles produced by HNA3 strain by SE with GC-MS

GC-MS identified six main semi-VOCs with a match factor ≥800, which do not exist in control samples (Table 2). Only 9-octadecenoic acid (z)- was identified in both HNA3 samples and control samples (Fig. S7 and S8). Phenol,2,4-bis(1,1-dimethylethyl) followed by 1,2-benzenedicarboxylic acid are the most abundant semi-VOCs identified in HNA3 with peak areas 37.55% and 14.15%, respectively.

**TABLE 2:** The main semi-VOCs identified in the extract of HNA3, retention time, area (%), match factor, molecular weight, molecular formula, CAS number, and if it was identified in control samples or not

No.	Compound name	Retention time (min)	Area (%)	Match factor	Molecular weight	Molecular formula	CAS no.	Identified in control
1	Methanamine, N-methoxy-	9.48	2.44	923	61	C_2_H_7_NO	1117-97-1	No
2	9-Octadecenoic acid (z)-	24.60	0.45	820	282	C_18_H_34_O_2_	112-80-1	Yes
3	1,2-Benzenedicarboxylic acid	29.47	14.15	960	390	C_24_H_38_O_4_	117-81-7	No
4	1h-purin-6-amine, [(2-fluorophenyl)methyl]	34.47	3.84	808	243	C_12_H_10_FN_5_	74421-44-6	No
5	Phenol,2,4-bis(1,1-dimethylethyl)-	35.51	37.55	999	206	C_14_H_22_O	96-76-4	No
6	2,3-bis[(trimethylsilyl)oxy]propyl (9z,12z)-9,12-octadecadienoate	36.25	0.68	805	498	C_27_H_54_O_4_Si_2_	54284-45-6	No
7	1h-indol-5-ol, 3-(2-aminoethyl) (serotonin)	36.45	1.38	869	176	C_10_H_12_N_2_O	50-67-9	No

### Identification of VOCs emitted from HNA3 strain using HS-SPME with GC-MS

Eight VOCs were identified in the headspace of HNA3 (Table 3) and do not exist in the headspace of the control sample (Fig. S9 and S10). All identified VOCs have a match factor ≥800. 9-Octadecenoic acid (z)-, methyl ester is the most common volatile compound emitted by HNA3 followed by hexadecanoic acid, methyl ester, and heptadecanoic acid, methyl ester with peak areas of 15.80%, 10.81%, and 1.99%, respectively.

**TABLE 3:** The main identified emitted-VOCs collected from headspace of HNA3, retention time, area (%), match factor, molecular weight, molecular formula, CAS number, and if it was identified in control samples or not

No.	Compound name	Retention time (min)	Area (%)	Match factor	Molecular weight	Molecular formula	CAS no.	Identified in control
1	Undecanoic acid, 10-methyl-, methyl ester	14.72	0.62	847	214	C_13_H_26_O_2_	5129-56-6	No
2	Dodecanoic acid	16.59	0.26	802	200	C_12_H_24_O_2_	143-07-7	No
3	Tetradecanoic acid, methyl ester	19.13	0.58	806	242	C_15_H_30_O_2_	124-10-7	No
4	Hexadecanoic acid, methyl ester	23.28	10.81	907	270	C_17_H_34_O_2_	112-39-0	No
5	9-Octadecenoic acid (z)-, methyl ester	26.57	15.80	931	296	C_19_H_36_O_2_	112-62-9	No
6	Eicosanoic acid, methyl ester	30.33	0.79	825	326	C_21_H_42_O_2_	1120-28-1	No
7	9,12-Octadecadienoyl chloride,(Z,Z)-	31.95	0.57	801	298	C_18_H_31_ClO	7459-33-8	No
8	Heptadecanoic acid, methyl ester	33.46	1.99	856	284	C_18_H_36_O_2_	1731-92-6	No

### Anti-fungal activity of individual pure VOCs and their mixture

Among the three pure VOCs that were evaluated for their anti-fungal activity, 9-octadecenoic acid (z)-, methyl ester and heptadecanoic acid, methyl ester demonstrated the ability to inhibit the fungal growth of all isolated phytopathogens, with varying degrees of inhibition (Fig. S11). Conversely, hexadecanoic acid, methyl ester did not exhibit any anti-fungal activity.

9-Octadecenoic acid (z)-, methyl ester exhibited strong inhibition of mycelial growth across all tested pathogenic fungi. Particularly, noteworthy was its potent anti-fungal effect against *Penicillium expansum*, with a substantial inhibition rate of 94.5% ± 0.6%. Additionally, it demonstrated notable inhibition percentages of 87.9% ± 0.4% against *Cladosporium cladosporioides*, and it effectively curtailed the growth of *Monilinia fructicola*, *Fusarium oxysporum*, and *Alternaria alternata*, achieving inhibition rates of 75.9% ± 0.5%, 74.2% ± 0.3%, and 56.2% ± 0.9%, respectively (*P* < 0.05). Heptadecanoic acid, methyl ester showed a moderate inhibitory effect recording inhibition percentages ranging from 81% ± 0.2% to 33% ± 0.6% (*P* < 0.05). Remarkable anti-fungal activity was observed when pathogenic fungi were treated with the mixture of VOCs [9-octadecenoic acid (z)-, methyl ester and heptadecanoic acid, methyl ester]. VOCs mixture recorded high inhibition percentage reached 100% ± 0.0% (*P* < 0.05) with *Alternaria alternata* and *Penicillium expansum*, followed by 98.2% ± 0.6%, 95.7% ± 1.2%, and 91.7% ± 1.0% (*P* < 0.05) with *Cladosporium cladosporioides*, *Fusarium oxysporum*, and *Monilinia fructicola*, respectively (Table 4).

**TABLE 4:** Anti-fungal effect of pure volatile organic compounds and their mixture on the growth of *Alternaria alternata*, *Penicillium expansum*, *Monilinia fructicola*, *Fusarium oxysporum*, and *Cladosporium cladosporioides* post-harvested phytopathogens^*a*^

Compound	*Alternaria alternata*		*Penicillium expansum*		*Monilinia fructicola*		*Fusarium oxysporum*		*Cladosporium cladosporioides*	
	Fungal diameter (mm)	Inhibition rate (%)	Fungal diameter (mm)	Inhibition rate (%)	Fungal diameter (mm)	Inhibition rate (%)	Fungal diameter (mm)	Inhibition rate (%)	Fungal diameter (mm)	Inhibition rate (%)
Control	85 ± 0.5^d^	0	60 ± 0.5^c^	0	88 ± 0.7^d^	0	75 ± 0.3^d^	0	63 ± 0.4^c^	0
9-Octadecenoic acid (z), methyl ester	40 ± 1.3^b^	56.2 ± 0.9^b^	8.0 ± 0.8^a^	94.5 ± 0.6^a^	25 ± 0.6^b^	75.9 ± 0.5^b^	23 ± 0.9^b^	74.2 ± 0.3^b^	12 ± 1.2^a^	87.9 ± 0.4^a^
Hexadecanoic acid, methyl ester	85 ± 0.2^d^	0	61 ± 0.6^c^	0	88 ± 0.9^d^	0	76 ± 0.9^d^	0	63 ± 0.9^c^	0
Heptadecanoic acid, methyl ester	58 ± 0.9^c^	33.7 ± 0.6^c^	15 ± 0.5^b^	81.8 ± 0.2^b^	38 ± 0.4^c^	60.2 ± 1.0^c^	42 ± 0.9^c^	44.2 ± 0.2^c^	24 ± 0.9^b^	67.2 ± 1.0^b^
VOCs mixture [9-octadecenoic acid (z)-, methyl ester + heptadecanoic acid, methyl ester]	5.0 ± 0.0^a^	100 ± 0.0^a^	5.0 ± 0.0^a^	100 ± 0.0^a^	10 ± 0.3^a^	91.7 ± 1.0^a^	8 ± 1.0^a^	95.7 ± 1.2^a^	6 ± 0.9^a^	98.2 ± 0.6^a^

^*a*^ The values represent the mean ± standard deviation (SD) where (*n* = 3). Letters in the same column (a, b, c, and d) indicate significant differences (*P* < 0.05) according to Tukey’s HSD test.

### Gene expression of representative secondary metabolite in HNA3 during fungi inhibition

**Fig 6:**
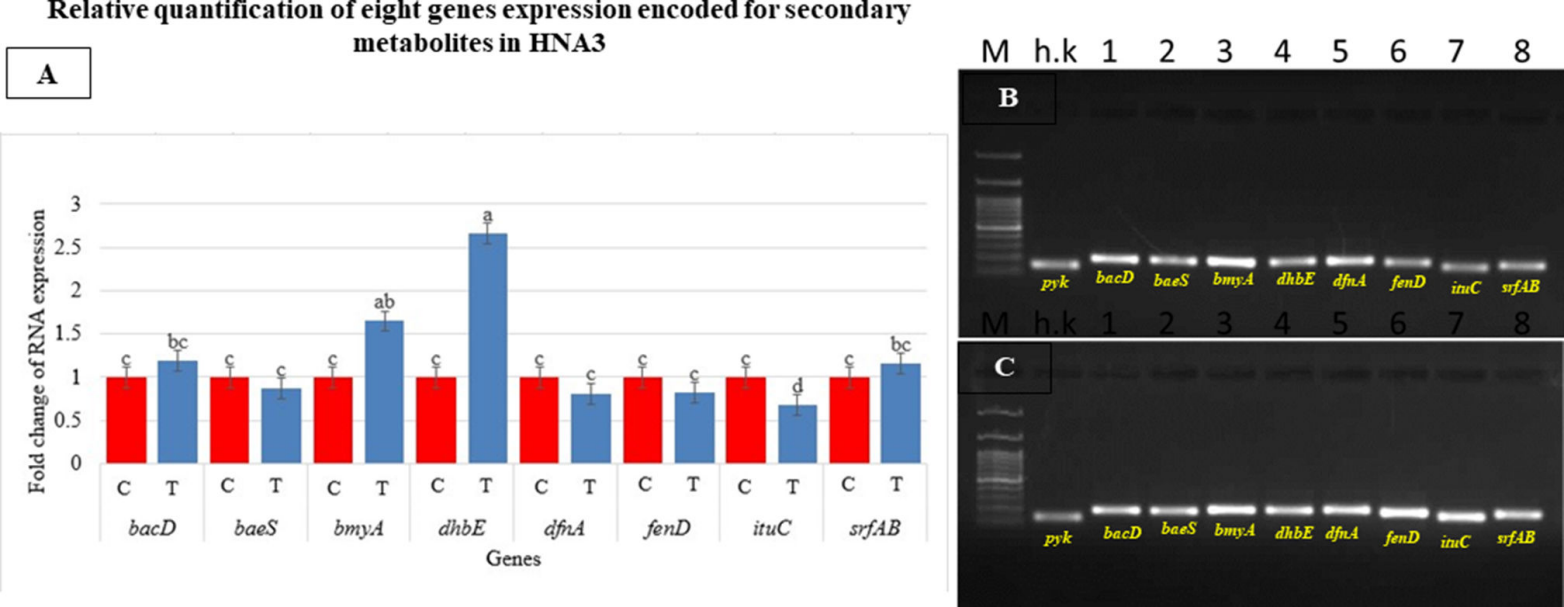
The relative quantification of expression for eight genes encoding secondary metabolites during the inhibition process in HNA3, as determined by real-time quantitative PCR (RT-qPCR). (A) The fold change in expression for *bacD*, *baeS*, *bmyA*, *dhbE*, *dfnA*, *fenD*, *ituC*, and *srfAB*. The data represent the mean ± SD with *n* = 9. Different letters “a, ab, bc, c, and d“ indicate statistically significant differences *(P* > 0.05) based on one-way ANOVA and Tukey’s HSD test. (B) The specificity of primers in the control sample. (C) The specificity of primers in the test sample, with each primer detecting its target gene (8 genes + h.k housekeeping gene) and forming an intact band in both control and test samples.

We detected eight genes that encode secondary metabolites in the genome of the HNA3 strain (Table 5) and investigated how their expression levels have been changed during the fungal inhibition stage. The expression of genes *dhbE*, *bmyA*, *bacD*, and *srfAB*, which encode bacillibactin, bacillomycin, bacilysin, and surfactin bioactive compounds, was all elevated at 2.6-, 1.6-, 1.19-, and 1.16-fold respectively, in comparison with the control. The expression of the genes *baeS*, *dfnA*, *fenD*, and *ituC*, which encode bacillaene, difficidin, fengycin, and iturin has no significant modification (Fig. 6).

**TABLE 5:** Primers of genes predicated to produce anti-fungal and plant growth-promoting secondary metabolites, gene function, encoded metabolites, biological function, and references

Gene	Primer ID	Primer sequence	Gene function	Metabolites	Biological function	References
*baeS*	*baeS*.F	GATGATTGAGCAGCTTCGCACC	Core biosynthetic gene of bacillaene	Polyketides bacillaene	Anti-fungal, induction of plant systemic resistance , and biofilm enhancers	(25, 26)
	baeS.R	GACCATTGCGTTTGTCCACACT				
*dif J*	dfnA.F	GCAGCTGAAACGTGATAAATCTCCT	Core biosynthetic gene of difficidin	Polyketides difficidin	Anti-fungal	(25, 26)
	dfnA.R	TTCCTTTCACCCACTGATTAAACGC				
*srfAB*	srfAB.F	ACCTGCCGAGAGACATGAGC	Core biosynthetic gene of surfactin	Non-ribosomal lipopeptides surfactin	Anti-fungal	(25, 26)
	srfAB.R	CCGAGGCCTTCTTGATCCGT				
*fenD*	fenD.F	CTCACTGCAAACGCGTTATGGAT	Core biosynthetic gene of fengycin	Non-ribosomal lipopeptides fengycin	Strong anti-fungal and induction (ISR)	(27, 28)
	fenD.R	GCGGTTATTCTCCAATGCGTTCA				
*bmyA*	bmyA.F	ATGAATGTTTGGAGAGCAGCACG	Core biosynthetic gene of bacillomycin	Non-ribosomal lipopeptides bacillomycin	Anti-fungal, induction (ISR), and biofilm formation	(29, 30)
	bmyA.R	AAGCTTGCGTCCGAATTTCTGTT				
*ituC*	ituC.F	CGTGACGCGCTGGACATTATC	Core biosynthetic gene of iturin	Non-ribosomal lipopeptides iturin	Anti-fungal	(29, 30)
	ituC.R	TGCTCTTCCATCCGCTTGACA				
*dhbE*	dhbE.F	TACAGCTTTCAGGAGGCAGTACG	Core biosynthetic gene of bacillibactin	Non-ribosomal lipopeptides bacillibactin	Siderophores promoting iron absorption and plant growth	(25, 26)
	dhbE.R	TCCGGCATATAAAGTTCCCAGCA				
*bacD*	bacD.F	GGAACGCCGCTCATTCTGAAG	Core biosynthetic bacilysin	Small-molecule peptide bacilysin	Anti-fungal	(31, 32)
	bacD.R	CAGTCGTCATACTCGCCCTGT				
*Pyk* (housekeeping)	Actin.F	GGC AGC TAT CGT TAC CCC TAC	Housekeeping gene	Pyruvate kinase	Reference gene	(33, 34)
	Actin.R	ACC GAA TAC AAG GCC GAG TTT				

## DISCUSSION

*Bacillus velezensis* possesses a rich and dynamic genetic content, resulting in the synthesis of a diverse array of valuable secondary metabolites, endowing it with multi-functional biological capabilities. In this study, five distinct post-harvest fungal strains were isolated and subsequently identified using a combination of morphological criteria (Fig. S1) and molecular characteristics (Table 1; Fig. S2 to S4). Fruits naturally contain a high percentage of moisture that makes them more susceptible to post-harvest rot by pathogenic fungi (6, 35). Most pathogenic fungi identified in this study have the capacity to produce mycotoxins, which pose a significant risk of causing serious diseases in both humans and animals (36 – 40). Furthermore, it is crucial to note that post-harvest losses of fruits and vegetables globally approach approximately 35%. This staggering statistic underscores the urgency for researchers to focus their efforts on identifying safe, readily available, and efficient biocontrol agents to mitigate these losses (6). Regular utilization of chemical fungicides contributes to the development of resistance in harmful fungi, rendering their control increasingly challenging (41). The isolation of similar post-harvest fungi has been observed in previous studies, which included the isolation of *Alternaria alternata* and *Cladosporium cladosporioides* from tomatoes (13, 37, 42), *Penicillium expansum*, from apple fruit (38, 43), *Monilinia fructicola* from peaches (44), and *Fusarium oxysporum* from potatoes (45).

*Bacillus velezensis* HNA3 strain inhibited the growth of all tested post-harvested fungal strains (Fig. 1). Also, VOCs produced by HNA3 showed significant anti-fungal activity reaching 100% inhibition rate with some fungal isolates (Fig. 2 and Fig. S5). As reported in previous studies, different *Bacillus velezensis* strains revealed strong biocontrol activity against many post-harvested phytopathogenic fungi (18). *Bacillus velezensis* g341 inhibited the growth of *Fusarium oxysporum* same as we observed in our study. However, HNA3 was able to inhibit the growth of *Fusarium oxysporum* using diluted bacterial culture reached 10^4^ in comparison to the g341 strain was used without dilution (46). *Bacillus velezensis* strain SL-6 was used in the biocontrol of *Penicillium expansum*, *Alternaria alternata*, and other post-harvested pathogens of pome fruits with inhibition percentages of 52% and 46%, respectively, while the diluted bacterial culture of HNA3 inhibited *Alternaria alternata* growth with a 47% inhibition percentage (47). Also, *Bacillus velezensis* AR1 showed anti-fungal activity against *Monilinia fructicola* phytopathogen (48). *Bacillus velezensis* OEE1 demonstrated inhibition of the pathogenic fungus *Cladosporium cladosporioides* with an inhibition percentage of 40%. While in this study, HNA3 exhibited a higher estimated inhibition percentage of 57% against *Cladosporium cladosporioides* (49).

In a similar study, VOCs emitted from *B. velezensis* XT1 suppressed the growth of *Monilinia fructicola* with an inhibition percentage of 37%. However, no inhibitory effect was observed on *Alternaria alternata* and *Fusarium oxysporum*. In contrast, in our current study, VOCs emitted by HNA3 demonstrated inhibition percentages of 22%, 100%, and 54% against *Monilinia fructicola*, *Alternaria alternata*, and *Fusarium oxysporum*, respectively (50). VOCs emitted by *Bacillus velezensis* BUZ-14 displayed an inhibition percentage of 6.0% against the growth of *Penicillium expansum*, while, HNA3-emitted VOCs yielded a significantly higher inhibition percentage of 48% against the *Penicillium expansum* phytopathogen (51). *Bacillus velezensis* acquires biocontrol capabilities likely due to the competitive pressures it faces within the rhizo-microbiome environment, thereby enhancing its prospects for survival. Its capacity to generate active compounds, particularly potent VOCs, enables it to effectively inhibit closely related species and various microorganisms within its ecological surroundings (52, 53). High vapor pressure and low molecular weight are characteristics of such VOCs, and from the perspective of control, these features increase the field of action, enhance membrane permeation, and ultimately increase the lethality of these bacteria (54). Also, as far as we know, this is the initial study to reveal that emitted VOCs most especially from the *Bacillus velezensis* strain can be used to control post-harvest *Cladosporium cladosporioides* completely, which is a benefit and an excellent precedent for future research.

HNA3 positively prompted the growth of the soybean plant and increased all plant parameters as well as the VOCs produced by HNA3 activated the germination of soybean seeds, enhanced root development, broke the dormancy of soybean seeds, and improved the growth of soybean seedlings (Fig. 3 and 4). Our results are in accordance with previous reports that *Bacillus* species particularly *Bacillus velezensis* strains have potential plant growth-promoting activity (55). Growth of soybean plants promoted after inoculating with *Bacillus velezensis* Ag75 (19) *Bacillus velezensis* S141 improved soybean growth and stimulated nodulation in the root (56). In addition, *Bacillus velezensis* CMRP 4490 enhanced the growth of soybean plants and improved seed germination (57). Overall, PGPR belonging to the *Bacillus* genus exhibits a multitude of mechanisms to facilitate plant growth. Some of these mechanisms exert a direct impact on the host plant, such as the synthesis of growth-promoting compounds. In certain instances, the effects are indirect, encompassing processes such as the breakdown of complex nutrients into forms more readily accessible to plant roots or the suppression of plant pathogenic organisms. These collective mechanisms collectively contribute to an environment conducive to enhanced plant growth (46, 58).

As mentioned in previous research, *Bacillus* spp. have the ability to produce VOCs that exhibit enhancing effects on different stages of plant growth (55). For instance, VOCs developed by *Bacillus amyloliquefaciens* L3 enhanced the growth of *Arabidopsis thaliana* plantlets (22). *Bacillus subtilis* SYST2 emitted VOCs that enhanced the growth of tomato plants (59). *Bacillus subtilis* AP-3 produced VOCs that promoted soybean plant growth and changed the architecture of the root system (60). However, this is the initial study to report the growth-promoting impact of VOCs developed by the *Bacillus velezensis* strain on soybean seedlings. Plants possess the capacity to detect microbial VOCs within their environment and promptly react to them, thereby influencing plant responses and regulating pivotal plant activities. Consequently, exploring the promotion of plant growth in reaction to microbial volatiles stands as a viable technique to enhance crop quality and production (61, 62). VOCs that elicit growth-promoting effects trigger an upregulation in the expression of genes encoding phytohormones in exposed plants. This in turn results in an elevation of endogenous phytohormones and ultimately leads to the enhancement of plant growth (62). Furthermore, VOCs emitted by *Bacillus* spp. can augment the growth of soybean plants by positively influencing root characteristics such as shape, elongation, and diameter. These improvements contribute to a heightened efficiency in water and essential nutrient absorption from the soil (60).

Microbial volatiles, including those produced by *Bacillus*, have exhibited their efficacy in enhancing seed germination of different plant species. For example, VOCs emitted from *Bacillus* sp. BCT9 caused an increase in root development during the seed germination stage of the cabbage plant (23). VOCs produced by *Bacillus* sp. MH778713 enhanced seed germination and broke the dormancy of *Prosopis laevigata* and *Arabidopsis thaliana* seeds same as we observed in our study (26). In addition, *Bacillus pseudomycoides* VOCs increased the shoot and root length of germinated seeds of wheat plants (30). Exposing seeds to *Bacillus* VOCs triggers a response within the seeds that leads to the regulation of endogenous phytohormone homeostasis, subsequently inducing cell division (63). No previous studies have monitored the effect of *Bacillus velezensis* volatiles on the germination of soybean seeds as we did in this study.

One of this study’s objectives is to look into the capacity of the HNA3 strain to release VOCs with anti-fungal and plant growth-promoting properties, as well as to find the best medium for producing the most effective volatile compounds by utilizing (TSA and 863A) growth media. As noticed from our results, TSA medium was the most suitable growth medium for HNA3 to synthesize effective VOCs with anti-fungal and plant growth-promoting properties (Fig. 2 and 4). Some researchers have confirmed the impact of the nutrients in growth media utilized by bacteria on the synthesis of VOCs (64). Gotor-Vila et al. (65), for example, found variations in VOC generation based on a culture medium (65). Our findings align with the observed anti-fungal effect of VOCs produced by *B. amyloliquefaciens* CPA-8 against phytopathogens such as *Monilinia laxa*, *M. fructicola*, and *Botrytis cinerea*. Importantly, this anti-fungal effect was notably more pronounced when *B. amyloliquefaciens* CPA-8 was cultivated in TSA medium, as opposed to nutrient agar supplemented with glucose (NAglu20) or nutrient yeast dextrose agar mediums (65). Even more, adding organic fertilizer to the growth media of two *B. amyloliquefaciens* strains showed differences in VOC production and response to *Ralstonia solanacearum* in tomatoes (66). Adding complex substrates to the medium, such as cellulose gum or carboxymethyl cellulose, significantly increased the anti-fungal activity of VOCs released by *B. subtilis* cultured on basic nutrition agar (67). The variability in VOC production among bacteria can be attributed to the composition of the growth media, specifically the presence of carbohydrates and proteins, as well as the metabolic capabilities of the bacteria themselves. Bacteria cultivated in media rich in proteins possess the capacity to generate VOCs endowed with anti-fungal properties. This phenomenon arises from the bacteria’s catabolic proficiency in breaking down proteins and utilizing amino acids as precursors for the synthesis of bioactive volatile compounds (51, 68). In our study, HNA3 produced anti-fungal VOCs after growing in protein-rich TSA growth media that contains high amounts of protein (17 g of casein peptone) and complex carbohydrates (soy peptone).

Indole acetic acid (IAA) is a hormone synthesized by plants, fungi, and bacteria. It plays a critical role in promoting plant growth and facilitating root development. Specific PGPRs are capable of producing IAA, which serves as a signaling molecule with significant implications for communication between plants and bacteria (69). HNA3 strain has the ability to produce IAA in small quantities in the existence and absence of tryptophan (precursor of IAA) after 10 days of incubation period in the dark at 37°C (Fig. 5 and Fig. S6). While *Bacillus velezensis* T13r strain produced IAA after 15 days of incubation period in the existence and absence of tryptophan (70), *Bacillus velezensis* FZB42 was only able to produce IAA in the presence of 5 mg/mL tryptophan (71). Bacterial growth parameters such as carbon supply, nitrogen source, oxygen presence, temperature, incubation duration, and bacterial population all play an important aspect in the process of IAA production. Therefore, there are no stable conditions for IAA indole production; it is depending on the growth factors for each bacterium (72). Generally, IAA is synthesized through two pathways tryptophan-dependent and tryptophan-independent. Tryptophan-dependent is the most common pathway that occurs in bacteria, where tryptophan is used as the precursor for IAA production through enzymatic transformation. In addition, some studies recorded the ability of bacteria including *Bacillus* spp. to produce IAA in the absence of tryptophan indicating that IAA can be produced in bacteria through tryptophan-independent as well (72, 73). We observed that HNA3 can produce a small quantity of IAA in the absence of an IAA precursor same as *Bacillus velezensis* T13r. A previous study demonstrated that, despite the targeted disruption of all putative genes associated with indole acetic acid (IAA) in the mutant strain *Bacillus velezensis* S141, the bacterium remained capable of producing IAA. This finding provides evidence that certain strains of *Bacillus velezensis* can produce IAA via a tryptophan-independent pathway, operating even in the absence of exogenous tryptophan (34).

We provided a GC-MS profile of the emitted VOCs and semi-VOCS compounds produced by HNA3. A total of 14 main VOCs have been identified. Six high molecular weight and less volatility organic compounds (semi-VOCs) have been identified in the ethyl-acetate extract of the HNA3 strain (Fig. S7 and Table 2), and eight low molecular weight and high volatility organic compounds (emitted VOCs) have been collected from the headspace of the HNA3 sample (Fig. S9 and Table 3). VOCs are organic compounds that evaporate at room temperature under standard pressure. They are classified into emitted VOCs that evaporate at a temperature less than 250°C and semi-VOCs that evaporate at a temperature greater than 260°C (74, 75). Previous studies have characterized the VOCs produced by various *Bacillus* strains through diverse extraction methods. A common approach is to employ a single extraction method. However, in our present investigation, we employed two distinct extraction methods, thereby enabling the identification of a more extensive range of bioactive compounds produced by HNA3.

HNA3 produces VOCs with a different chemical structure including (1) fatty acids (dodecanoic acid), (6) fatty acid, methyl ester (FAME) [(tetradecanoic acid, methyl ester), (hexadecanoic acid, methyl ester), (9-octadecenoic acid (z)-, methyl ester), (eicosanoic acid, methyl ester), (heptadecanoic acid, methyl ester), and (undecanoic acid, 10-methyl-, methyl ester)], (1) phenols [phenol,2,4-bis(1,1-dimethylethyl)], (1) benzene derivatives (1,2-benzenedicarboxylic acid), (1) indoles [1h-indol-5-ol, 3-(2-aminoethyl)] or serotonin, (2) amines methanamine and [1h-purin-6-amine,(2-fluorophenyl)methyl], and (2) organic acid derivatives [2,3-bis (trimethylsilyl)oxy-propyl(9z,12z)-9,12-octadecadienoate] and [9,12-octadecadienoyl chloride,(Z,Z)-]. We will direct the discussion to the most detected VOCs compounds.

Phenol,2,4-bis(1,1-dimethylethyl) and 1,2-benzenedicarboxylic acid are the most abundant semi-VOCs with peak areas of 37.55% and 14.15%, respectively. While 9-octadecenoic acid (z)-, methyl ester is the most common volatile compound emitted by HNA3 followed by hexadecanoic acid, methyl ester and heptadecanoic acid, methyl ester with peak areas 15.80%, 10.81%, 1.99%, respectively.

Our results are in accordance with a previous study that reported the ability of *Bacillus velezensis* ZSY-1 strain to produce phenol,2,4-bis(1,1-dimethylethyl) with significant anti-fungal activity against phytopathogenic fungi *Botrytis cinerea* and *Alternaria solani* (76). Ramírez et al. confirmed that the 2,4-di-tert-butylphenol compound produced by *Bacillus* sp. MH778713, which is synonymous with phenol,2,4-bis(1,1-dimethylethyl), can break the dormancy of *Prosopis laevigata* and *Arabidopsis thaliana* seeds and stimulates seed germination under chromium stress (26). Additionally, phenol,2,4-bis(1,1-dimethylethyl) was detected in the extract of PGPR *Bacillus velezensis* WSW007 which stimulates tobacco plant growth and upregulates the expression of genes related to plant development (77). Drawing from previous research, it is possible that phenol,2,4-bis(1,1-dimethylethyl) plays a significant role in the bioactivity of VOCs produced by HNA3. These potential roles include disrupting the dormancy of soybean seeds, augmenting seed germination, promoting soybean growth, and exerting anti-fungal effects.

*Bacillus cereus* synthesizes 2-benzenedicarboxylic acid, which exhibits inhibitory activity against *Sclerotinia sclerotiorum* (78). Besides, it was identified in *Bacillus atrophaeus* XEGI50 extract that was used as a biocontrol agent against verticillium wilt disease in cotton plants (79). Moreover, *B. subtilis* KA9 produces 1,2-benzenedicarboxylic acid and other benzene derivatives active compounds. KA9 induces chili plant development and increases plant resistance against bacterial wilt disease (80). 1,2-Benzenedicarboxylic acid was not identified in *Bacillus velezensis* strains before.

9-Octadecenoic acid (z)-, methyl ester and hexadecanoic acid, methyl ester are produced by different *Bacillus* spp. As an instance, Selvamani et al. identified octadecanoic acid (z)-, methyl ester and hexadecanoic acid, methyl ester as active constituents in the methanol fraction of *Bacillus amyloliquefaciens* (Ba-14.5). Ba-14.5 exhibits nematicidal activity according to their report (81). Moreover, Keerthana et al. identified a high area percentage of octadecanoic acid (z)-, methyl ester (31.1%) and hexadecanoic acid, methyl ester (14.1%) in the crude extract of *Bacillus amyloliquefaciens* (sic.). Such strain has anti-fungal and plant growth-promoting activity (82). Octadecanoic acid (z)-, methyl ester was also detected in *Bacillus atrophaeus* XEGI50 (79) and *Streptomyces alfalfae* XN-04 (83). There are no prior studies on the production of heptadecanoic acid, methyl ester by *Bacillus* strains. However, it was detected by GC-MS emitted from *Trichoderma viride*, which is used as a chickpea seed protectant against Fusarium wilt disease (84).

We selected the three predominant pure VOCs emitted by HNA3 [9-octadecenoic acid (z)-, methyl ester, hexadecanoic acid, methyl ester, and heptadecanoic acid, methyl ester] for evaluating their anti-fungal activity against the five isolated post-harvest phytopathogens. Our selection for that volatile compounds is based on the limited available information regarding their anti-fungal effects. Both pure 9-octadecenoic acid (z)-, methyl ester and pure heptadecanoic acid, methyl ester suppressed the mycelial growth of all phytopathogenic fungi at varying inhibition rates (Fig. S11 and Table 4). On the other hand, fungal growth was not affected by pure hexadecanoic acid, methyl ester. Our outcomes are consistent with a prior study that demonstrated the anti-fungal effect of standard methyl oleate [synonyms of 9-octadecenoic acid (z)-, methyl ester] against four human fungal pathogens, namely *Paracoccidioides brasiliensis*, *Candida albicans*, *Candida gattii*, and *Candida neoformans* (85). This is the inaugural study that demonstrates the pure 9-octadecenoic acid (z)-, methyl ester anti-fungal effect against plant phytopathogenic fungi.

Heptadecanoic acid, methyl ester showed moderate inhibitory action against all tested phytopathogenic fungi. As an initiatory study, this research confirmed the anti-fungal activity of volatile heptadecanoic acid, methyl ester.

Hexadecanoic acid, methyl ester was detected by Rajaofera et al., as an active volatile compound in the culture of *Bacillus atrophaeus* HAB-5 and the same study reported that pure hexadecanoic acid, methyl ester displayed weak inhibitory activity (10.83%) against *Colletotrichum gloeosporioides* pathogenic fungi (86). While pure hexadecanoic acid, methyl ester did not show anti-fungal activity against fungi used in this study, phytopathogenic fungi sensitivities vary to the same compound according to the fungi’s virulent capacity and their ability to detoxify this compound (87).

In our study, the most detected compounds in the headspace of HNA3 are FAMEs including 9-octadecenoic acid (z)-, methyl ester and heptadecanoic acid, methyl ester. FAMEs are known to have anti-fungal activity due to their detergent and melting effect on the plasma membrane of the fungal cells leading to cell lysis and death (81). Other studies have proposed that FAMEs contribute to an elevation in reactive oxygen species (ROS) within fungal cells due to the membrane desaturation process. As a result of this elevated ROS level, the normal functions of the mitochondrial membrane within the fungal cells are disrupted (88). Moreover, there is another proposed mechanism that FAMEs can block the enzymes involved in essential cellular processes such as DNA replication and nutrient metabolism (89).

To explore the possibility of improving the inhibitory efficacy against phytopathogenic fungi, we examined the inhibitory effect of the pure VOCs mixture [9-octadecenoic acid (z)-, methyl ester + heptadecanoic acid, methyl ester]. The mixture of pure VOCs displayed significant inhibitory effects as they completely forbade the mycelial growth of most phytopathogenic fungi. It is worth mentioning that no previous study investigated the anti-fungal potential of the [9-octadecenoic acid (z)-, methyl ester + heptadecanoic acid, methyl ester] mixture. Our results of the VOCs mixture effectiveness are in accordance with an earlier study that demonstrated that a mixture of volatile organic acids produced by *Lactobacillus sanfrancisco* CB1 has a synergistic action to suppress mold growth (90). In addition, vapors from acetic acid and propionic acid mixture showed promising antibacterial activity (91). VOCs are chemical compounds with diverse and intricate chemical structures, resulting in various modes of action against pathogens. Their inhibitory effects often stem from synergistic interactions among the compounds, rather than relying solely on the properties of individual chemicals (92). This explains the effective ability of these volatile substances emitted by the HNA3 strain to prevent the growth of some pathogenic fungi completely.

We discovered that HNA3 is able to produce two types of indole compounds under different growth conditions (serotonin and IAA). Serotonin [1h-indol-5-ol, 3-(2-aminoethyl)] was detected by GC-MS after 2 days of incubation using tryptone soy broth (TSB) medium with 1.38% area percentage, while IAA was detected using colorimetric method after 10 days of incubation in the dark using nutrient broth (NB) medium, as we mentioned before. Perhaps we need more studies and research on the pathways used by HNA3 to produce those two types, but we have confirmed the fact about its capability of producing indoles, which are used as signal VOCs between microbial communities (93) Furthermore, these indole compounds play a crucial role in promoting plant root development by influencing and modulating root architecture (94, 95). Our results are in line with a previous study that stated that endophytic *Bacillus amyloliquefaciens* SB-9 produced serotonin as an intermediate for melatonin production (96). In addition, endophyte *Pseudomonas fluorescens* RG11 produced serotonin in the melatonin pathway and promoted the root development of a grape plant (97)

In our previous work, we identified 12 biosynthetic gene clusters in the HNA3 genome using bioinformatics analysis (17). In the current study, we conducted real-time quantitative PCR (RT-qPCR) on the genes associated with the production of secondary metabolites for a better understanding of the non-volatile active compounds (lipopeptides and polypeptides) that could be expressed in HNA3 and expected to be secreted (Table 5). The results indicated that the genes *dhbE*, *bmyA*, *bacD*, and *srfAB* which encode the bioactive substances bacillibactin, bacillomycin, bacilysin, and surfactin, respectively, are highly expressed (Fig. 6). Bacillibactin encourages iron absorption and plant development (25), while bacillomycin has the bioactivity of anti-fungal stimulation of phyto-resistance and modulation of biofilm formation (29). Surfactin and bacilysin are both known to have anti-fungal activity (25, 98). Our findings are consistent with a prior study that conducted a comparative analysis of four distinct *Bacillus velezensis* strains originating from diverse habitats. That study revealed that *Bacillus velezensis* UCMB5113 and At1, which thrive close to plant roots, exhibit the capability to express genes responsible for encoding lipopeptides antibiotic compounds. The capacity to secrete lipopeptide antibiotics confers an advantageous trait upon PGPRs owing to their root-associated lifestyle (99). Similarly, *Bacillus velezensis* 2211 strain upregulates the expression of *dhbE* and *bmyA* encoding bacillibactin and bacillomycin (100). In addition, surfactin biosynthetic genes expressed in biocontrol agent *Bacillus velezensis* T149-19 (28) and *bacD* gene expression increased in biocontrol *Bacillus velezensis* SBB (98). The regulation of gene expression associated with antimicrobial compounds during the inhibition phase against pathogens signifies a primary line of defense for bacteria. This mechanism is likely to play a crucial role in maintaining the bacteria’s survival and competitive advantage within its environment (32).

### Conclusion

*Bacillus velezensis* HNA3 emerges as a PGPR, characterized by its capacity to synthesize a diverse range of bioactive compounds. These encompass fatty acids, fatty acids methyl esters, phenols, benzene derivatives, organic acids, indoles, and amines. Furthermore, HNA3 exhibits an upregulated expression of genes responsible for synthesizing lipopeptides namely, bacillibactin, bacillomycin, bacilysin, and surfactin. The volatiles emitted by HNA3 significantly inhibited the growth of five distinct post-harvest fungal isolates, including *Alternaria alternata*, *Cladosporium cladosporioides*, *Fusarium oxysporum*, *Penicillium expansum*, and *Monilinia fructicola.* Beyond its role in promoting soybean plant growth, HNA3-emitted VOCs also demonstrate the capacity to enhance seed germination. TSA media appear as the optimal substrate for HNA3, facilitating the production of potent VOCs with anti-fungal and plant growth-promoting activities. It is noteworthy that the individual volatile compounds, 9-octadecenoic acid (z)-, methyl ester and heptadecanoic acid, methyl ester, exhibit remarkable inhibitory effects against the growth of all isolated phytopathogens. Intriguingly, their combined application synergistically strengthens the efficacy of fungal growth inhibition. In addition, HNA3 produced indole acetic acid and serotonin, both recognized as indoles with the potential to promote plant growth. This comprehensive investigation underscores the multifaceted bioactivity of HNA3, solidifying its potential as an impactful biocontrol and plant growth-enhancing agent.

## MATERIALS AND METHODS

### Growth condition and culture media for *Bacillus velezensis* HNA3

HNA3 strain was isolated by our group from the rhizosphere region of the soybean plant in 2013. Xu et al. reported HNA3 strain morphology characteristics, examined the anti-fungal activity of the HNA3 broth culture against some soil-borne phytopathogenic fungi, proposed its biocontrol activity to the presence of lipopeptides compounds in its broth culture, presented the most favorable cultivation conditions of HNA3, and genetically identified as *Bacillus amyloliquefaciens* based on morphological characteristic and 16S rRNA sequence analysis (101). In 2022, based on genetic and bioinformatics studies, the HNA3 strain was reclassified as the *Bacillus velezensis* strain according to the whole genome sequence and phylogenetic studies. It was deposited in NCBI database with accession number CP040881. Zaid et al. reported HNA3 whole genomic feature, identified newly Lanthipeptide biosynthetic gene cluster that was exclusively present in the HNA3 strain, and predicated the genetic insights of biocontrol and plant growth-promoting activity of HNA3 strain (17).

*Bacillus velezensis* HNA3 strain was isolated and kept in our lab “Key Laboratory of Agricultural Microbiology, Huazhong Agricultural University”. A loopful of HNA3 glycerol stock was streaked over a freshly prepared TSA plate (17 g/L casein peptone, 3 g/L soya peptone, 2.5 g/L glucose monohydrate, 5 g/L sodium chloride, 2.5 g/L dipotassium phosphate, and 15 g/L agar) and incubated at 37°C for 48 hours. A single bacterial colony was transferred to 5 mL of TSB and incubated in a shaker incubator at 37°C with 100 rpm for 24 hours. The concentration of bacterial culture was adjusted and employed according to the conditions of each experiment and validated by counting bacterial colonies on TSA for 24 hours at 37°C.

### Isolation and identification of post-harvested fungi

Rotten fruit and vegetable samples (tomato, apple, peach, and potato) were collected randomly from Cairo, Egypt, and kept separately in a sterile plastic bag at 4°C until the next step. Lesion parts of the fruit or vegetables were inoculated on the surface of potato dextrose medium PDA (200 g/L potato infusion, 20 g/L dextrose, and 15 g/L agar) plates and then incubated at 25°C for 7 days. The isolated fungi were morphologically evaluated by observing colony features, including shape, size, color, texture, and hyphae. The microscopic assessment was conducted by examining small samples of mycelium, spores, or conidia using a compound microscope in conjunction with a digital camera (102). To confirm the findings of morphological and microscopic identification of isolated pathogenic fungal strains and to identify them at the species level, they were subjected to the identification of ITS or 18S ribosomal RNA (18S rRNA) genetic sequences analysis by using ITS1 and ITS2 rDNA or NS1 and NS25 primers (103).

DNA extraction and PCR for the isolated phytopathogen were accomplished following the method outlined by Al Jaradi et al.; specifically, fungal mycelia (50 mg) were harvested from mature fungal cultures grown on PDA. The mycelia were then ground, and 600 µL of lysis buffer was added. The resulting mixture was incubated at 65°C for 1 hour. Subsequently, an equal volume of phenol:chloroform:isoamyl alcohol (25:24:1) was introduced, followed by centrifugation at 10,000 *g* for 15 minutes. DNA precipitation was achieved by adding 10 µL of sodium acetate and 180 µL of isopropanol to the supernatant, which was then incubated at −20°C for 20 min. After centrifugation at 10,000 *g* for 2 minutes, the resulting pellets were washed with 70% ethyl alcohol and allowed to air dry. These dried pellets were resuspended in 100 µL of Tris-EDTA buffer (10 mM Tris HCL and 1 mM EDTA) and stored at −20°C until further use. Quantification of DNA samples was performed using a Nanodrop spectrophotometer (104). Purified PCR samples were sent to Macrogen Biotechnology Company for genomic sequencing services (Macrogen, Teheran-ro 238, Gangnam-gu, Seoul, Republic of Korea). To determine the phylogenetic position of the isolated phytopathogens, we used the NCBI BLAST query tool to find the most likely sequences identical to the acquired sequences. MEGA software’s MUSCLE alignment tool was used to align the identical sequences. The maximum likelihood trees were constructed with 1,000 bootstrap replicates based on the Kimura 2-parameter model (105).

Pathogenicity test was performed on healthy, mature fruits and vegetables frequently using the method of inoculation by a wound of fruits and vegetables to promote infection and spore formation (106).

### Anti-fungal activity of HNA3 against post-harvested phytopathogens

Using the dual culture technique, a fungal plug measuring 5 mm in diameter, taken from an actively growing fungal culture, was positioned along one edge of a fresh PDA plate. On the opposing side of the plate, 50 µL of HNA3 broth culture at a concentration of 1 × 10^4^ CFU/mL was applied onto a 5-mm filter paper. The control plate consisted of a PDA plate solely inoculated with the phytopathogens. Subsequently, all the plates were placed in an incubator set at 37°C for 24 hours to reach the bacterial stationary phase. This was followed by continuous incubation at 25°C to facilitate fungal growth until the mycelia completely covered the surface of the control plate. Each treatment was replicated three times to ensure the robustness of the results. This experimental procedure was repeated for all five fungal isolates (22), and the inhibition percentage was calculated according to the following formula:


$$Inhibition\;percentage(\%)=\frac{D_{i}}{D}\times 100\%,$$

where *D*_*i*_ is the distance of the inhibition zone in mm and *D* is the distance between the pathogenic fungi and central HNA3 disc (107).

### Anti-fungal activity of emitted volatiles produced by HNA3

A double petri dish test was employed to quantify the inhibitory potential of HNA3-emitted VOCs (108). For this experiment, two different culture media were employed: TSA and 863A (10 g/L peptone, 10 g/L yeast extract, 20 g/L D-glucose, and 15 g/L agar-agar) for the growth of HNA3 bacteria, while PDA was used to facilitate fungal growth. To establish a sterile space where the pathogenic fungus could be exposed to VOCs produced by HNA3, a fungal growth plug with a diameter of 5 mm was centrally placed on a freshly prepared PDA plate. On the freshly prepared TSA and 863A plates, 100 µL of HNA3 broth culture (1 × 10^8^ CFU/mL) was evenly smeared across the plate surface. The lids of these inoculated plates were exchanged with the base of a PDA plate containing a fungal plug. Both base plates were hermetically sealed using parafilm to prevent the escape of VOCs. Control plates were also established utilizing TSA and 863A plates that were devoid of any bacterial inoculum. Subsequently, the paired plates were incubated at 37°C for 24 hours, followed by an additional incubation period at 25°C until the mycelia of the tested phytopathogen fully covered the surface of the control plates. Each treatment was subjected to replication three times, and the experimental procedure was repeated for all five fungal isolates. The width of fungal growth was measured after the incubation period. The observed outcomes were calculated as a percentage of inhibition and compared to the control. The inhibition percentage was calculated according to the following formula (87):


$$Inhibition\;percentage\;(\%)=\frac{\left(D_{C}-D_{T}\right)}{\left(D_{c}-5mm\right)}\times 100,$$

where *D*_*C*_ represents the width (mm) of the phytopathogen in the control plate, and *D*_*T*_ represents the width (mm) of the phytopathogen in the treatment plate.

### Plant growth-promoting effect of HNA3

We investigated the influence of HNA3 broth culture on the growth of soybean plants (Zhong Huang 13 soybean seeds) (109). Soil samples were collected from Huazhong Agriculture University, Hubei Province, China. The experiment comprised two main treatments: soil + soybean seeds (control) and soil + soybean seeds + HNA3. Plastic pots with a capacity of 500 g were filled with the collected soil, and each pot was transplanted with 10 Zhong Huang 13 soybean seeds, and each treatment had six replicates. To prepare the HNA3 inoculum, a single colony of HNA3 was transferred to a 5-mL sterilized TSB medium. The culture was then incubated at 37°C and 100 rpm for 24 hours. Once the soybean seeds had germinated and developed their first true leaves, an individual germinated seed was chosen for cultivation in each pot. This selection process ensured that all the germinated seeds chosen had uniform lengths. Consequently, every treatment was subjected to six replicates. In the treatments where inoculation was applied, 1 mL of the HNA3 inoculum (1 × 10^4^ CFU/mL) was introduced into the soil in close proximity to the primary root of the germinated seeds. In the control group, germinated seeds were cultivated under identical conditions but without the introduction of HNA3 inoculum. Soybean plants were cultivated within a controlled light chamber with a light/dark cycle of 16 hours of light and 8 hours of darkness, maintained at a temperature of 30°C ± 2°C. After a growth period of 30 days, the plants were harvested for assessment. Parameters evaluated included fresh weight, shoot length, root length, and the extent of root system branching (110).

### Seed germination and seedling growth-promoting effect of VOCs produced by HNA3

To assess the impact of emitted volatiles on soybean seed germination, a plate assay was employed. The process began by sterilizing the surfaces of the soybean seeds. This was achieved by immersing the seeds in a solution of 10% NaClO (900 mL of sterile distilled water + 100 mL of commercial bleach) for a duration of 4 minutes. Following this step, the seeds were subjected to a 2-minute immersion in 70% ethyl alcohol. The final rinse consisted of a 1-minute treatment with sterile distilled water. The sterilized seeds were then drained within a laminar flow sterile hood. In the plate assay, a volume of 100 µL of HNA3 broth culture (1 × 105 CFU/mL) was inoculated onto small-sized plates (35 mm) containing sterilized TSA and 863A media. These plates were then incubated at 37°C for 24 hours. Sterilized seeds were meticulously transferred to one side of larger plates (200 mm) containing freshly prepared and sterilized agar (15%). On the opposite side of this plate, a previously prepared and inoculated TSA or 863A plate (35 mm) was positioned. For comparison, control plates were prepared where sterilized seeds were placed onto agar plates without any bacterial inoculum. In order to ensure the reliability of the results, each treatment condition had six replicates. The whole system was wrapped tightly and then incubated for a duration of 15 days within a light chamber, maintaining a light/dark cycle of 16 hours light and 8 hours darkness at a temperature of 30°C ± 2°C, to facilitate seed germination (111). To calculate the germination percentage (GP) we used the following formula: GP = Number of germinated seeds/Total seeds used × 100 (112).

As indicated by Park et al. (113) with some modifications, we initiated the preparation of TSA and 863A media and subsequently poured 25 mL of the prepared media into individual glass jars. These glass jars containing the media were securely sealed and subjected to sterilization at 121°C for a duration of 30 minutes. Prior to the complete solidification of the sterilized media, 100 µL of HNA3 liquid culture (1 × 10^5^ CFU/mL) was thoroughly mixed with the molten sterilized media in the jars. The resulting mixture was then incubated at 37°C for 24 hours to encourage the growth of the bacteria.

Uniformly sized germinated soybean seeds were transferred to plastic cups, each containing 200 g of sterilized soil. To facilitate exposure to the emitted VOCs, holes were introduced at the bottom of these plastic cups. Then, these cups were carefully fixed onto the glass jars containing the inoculated media. In order to prevent the release of VOCs produced by HNA3, both the plastic cups and the glass jars were meticulously coated with parafilm. As a control measure, plastic cups containing germinated soybean seeds with holes were affixed to empty jars. The cups attached to the jars were allowed to undergo growth for a period of 15 days within a controlled light chamber. The chamber maintained a light cycle of 16 hours and a darkness cycle of 8 hours, maintaining a temperature of 30°C ± 2°C. Each treatment had six replicates. The assessment of seedling growth enhancement was based on the observed differences in terms of fresh weight, root length, and shoot length between the control samples and the seedlings exposed to the VOCs emitted by HNA3 (113).

### Qualitative and quantitative estimation of IAA

The spectrometric procedure was chosen to assess and quantify HNA3’s capability to synthesize IAA. A volume of 1 mL of HNA3 broth (1 × 10^8^) was inoculated into 50 mL of Nutrient Broth (NB) medium (3 g/L beef, 5 g/L peptone, and 8 g/L NaCl). This mixture was supplemented with different concentrations of tryptophan (0, 0.2, 0.5, 2, and 5 mg/mL). The culture was incubated in darkness for a period of 14 days at a temperature of 37°C. On the 2nd, 6th, 10th, and 14th days, 10 mL of inoculation cultures was collected and centrifuged at 4,000 rpm for 30 minutes. A volume of 2 mL of supernatant was incubated in the dark for 25 minutes with one drop of orthophosphoric acid and 4 mL of Salkowski’s reagent (300 mL concentrated sulfuric acid:500 mL d.H_2_O:15 mL 0.5 M FeCl_3_). The developed pink or red color designated the successful IAA production. A UV-visible spectrophotometer set to 530 nm was used to measure the intensity of the developed color. The experiment was replicated three times to verify the concentration of IAA. To determine the optimal tryptophan concentration for IAA formation, the quantity of IAA was calculated from the standard IAA curve (70). For the standard curve, different concentrations of indole acetic acid were prepared and treated with Salkowski’s reagent, the developed color was measured with a UV-visible spectrophotometer set to 530 nm, and the results were represented in a curve.

### GC-MS analysis of semi-volatiles produced by HNA3 using ethyl acetate extraction

#### Sample preparation

To identify the semi-VOCs produced by HNA3, a 48-hour HNA3 broth culture was precipitated by centrifuging at 400 rpm for 30 minutes, the supernatant was collected and mixed with ethyl acetate, as a solvent, at a 1:1 (vol/vol) ratio. Through vigorous shaking for 50 minutes, the mixture was partitioned into an ethyl acetate phase and an aqueous phase. The extract was concentrated by evaporating the ethyl acetate at 50°C in a rotary evaporator (11). The experiment was designed in triplicate, and the controls were ethyl acetate extract of TSB media without bacteria.

### GC-MS analysis of emitted VOC using HS-SPME

#### Sample preparation

Headspace vials (20 mL) were filled with 3 mL of melted TSA culture media. The TSA-filled vials underwent a 30-minute sterilization process at 121°C. The surface of the culture medium in the sample vials was then injected with 100 µL of HNA3 cell suspension (1 × 10^7^ CFU/mL). The vials were incubated for 4 days at 37°C. The experiment was designed in triplicate, and the control samples consisted of vials that were not inoculated and contained only TSA media. SPME fiber coated with DVB/CAR/PDMS (divinylbenzene/carboxen/polydimethylsiloxane) was used. Vials of the samples were immersed in a water bath to equilibrate for 27 minutes at 74°C, and SPME fibers were exposed to the vial’s headspace for 53 minutes. The GC injector desorbed the trapped chemicals for 4 minutes at 250°C (114).

#### Device information

The Trace GC-TSQ mass spectrometer (Thermo Scientific, Austin, TX, USA) with a direct capillary column TG-5MS (30 m × 0.25 mm × 0.25 m film thickness) was used for the experiment. The temperature of the column oven was initially held at 50°C, then increased by 5°C/minute to 250°C and maintained for 2 minutes before increasing by 30°C/minute to the ultimate temperature of 300°C and held for 2 minutes. The injector and MS transfer lines were held at steady temperatures of 270°C and 260°C, respectively, and helium was used as a carrier gas at a steady rate of flow of 1 mL/minute. The solvent latency was 4 minutes, and diluted samples were injected automatically using an auto-sampler AS1300 coupled to a GC in split mode. EI mass spectra were collected in full scan mode at 70 eV ionization voltages over the *m*/*z* 50–500 range. The ion source’s temperature was set at 200°C. The various compounds were identified by matching their mass spectra to the WILEY 09 and NIST 14 mass spectral databases. According to the National Institute of Standards and Technology (NIST), a match factor of 900 or above is considered excellent, 800–900 is considered good, 700–800 is considered fair, and 600 is considered bad. Main identified compounds with a match factor ≥800 have been considered, while compounds with less than 800 have been neglected.

### Anti-fungal activity of individual pure VOCs

Based on the GC-MS analysis, the most prevalent volatiles collected from the headspace of the HNA3 sample were identified as 9-octadecenoic acid (z)-, methyl ester, hexadecanoic acid, methyl ester, and heptadecanoic acid, methyl ester. However, due to the lack of comprehensive evaluation regarding their anti-fungal activity, we proceeded to investigate the inhibitory potential of these individual compounds against the five isolated post-harvest phytopathogens. The three pure volatile compounds were purchased from MilliporeSigma Company (Massachusetts, USA): 9-octadecenoic acid (z)-, methyl ester (CAS no. 112-62-9 and purity 70%), hexadecanoic acid, methyl ester (CAS no. 112-39-0 and purity ≥99.0%), and heptadecanoic acid, methyl ester (CAS no. 1731-92-6 and purity ≥99.0%).

A fungal plug of an actively growing fungus with a 5-mm diameter was introduced in the midpoint of a freshly prepared PDA plate. A concentration of 100 µL/L of individual pure VOCs has been spotted on sterilized filter paper (20-mm diameter). Then, the spotted filter paper was placed in the center of the PDA plate lid. In the control plate, filter paper was spotted with sterilized distilled water. The plates were sealed tightly with parafilm to prevent VOC escape and then incubated at 25°C. Results were obtained after the fungus growth covered the surface of the control plate. Each treatment was tested out three times. The same procedures were accomplished for all of the five fungal isolates. The percentage of inhibition was calculated in comparison to the control as mentioned before (87).

### Anti-fungal activity of VOCs mixture

Based on the findings of the previous experiments, it was observed that 9-octadecenoic acid (z)-, methyl ester and heptadecanoic acid, methyl ester possess anti-fungal activity against all the tested phytopathogens. To assess the inhibitory effect of a mixture comprising these pure volatile compounds, we replicated all the procedures of the earlier experiment. However, the filter paper was impregnated with a mixture of volatile compounds at a concentration of 100 µL/L. This mixture consisted of 50 µL/L of 9-octadecenoic acid (z)-, methyl ester and 50 µL/L of heptadecanoic acid, methyl ester. The control was the same as the previous experiment. Following the incubation period, the outcomes were recorded, and the inhibition percentage was calculated, employing the same methodology as the previous experiment (87).

### Detection and quantification of secondary metabolite genes expression in HNA3 using RT-qPCR

A freshly cultured fungal plug, with a diameter of 5 mm, was positioned at the center of a PDA plate. A volume of 100 µL of HNA3 broth culture was introduced into the plate, at a distance of 2.5 cm from the fungal plug. The plate was then subjected to an incubation period of 24 hours at a temperature of 37°C, followed by an additional incubation period of 7 days at 25°C. For comparative purposes, a PDA plate was inoculated solely with HNA3 broth culture and utilized as the control in this experiment (98). For total RNA extraction, HNA3 colonies were scratched from the interfering inhibition zone directly from agar growth plates. Following the manufacturer’s guidelines, total RNA was extracted and purified from bacterial colonies using the Qiagen RNeasy Mini Kit cat# 74104. Quantification and quality assessment of RNA samples have been done using Nanodrop spectrophotometer at A230, A260, and A280. First strand cDNA synthesis has been done for RNA samples using the Promega cDNA Synthesis AMV Reverse Transcriptase Kit cat# M5108 according to the manufacturer’s instructions. cDNA synthesis was performed on Bio-Rad100 Thermal cycler from treatment plates and control plates (115). RT-qPCR was used to investigate the eight secondary metabolite genes (*baeS*, *dfnA*, *srfAB*, *fenD*, *bmyA*, *ituC*, *dhbE*, and *bacD*) detected in HNA3 genome through synthesized cDNA. Housekeeping gene *pyk* was used for internal control for gene expression (33). Primer design for genes under study was accomplished using primer3plus software (Table 5). The reaction temperature was set at 95°C for 3 minutes, followed by 40 cycles of 95°C for 5 seconds, followed by 60°C–65°C for 20–30 seconds. The experiment was run on a Bio-Rad cfx96 (Applied Biosystems, Foster City, California, USA), and 2 − ∆∆Ct approach was used for calculating fold gene expression (98). The experiment was run three times for each treatment and performed in triplicate.

### Statistical analysis

We used IBM SPSS Statistics software version 26 to calculate the one-way analysis of variance and Tukey’s honestly significant difference test (*P* < 0.05).

## Data Availability

Whole genome sequences of *Bacillus velezensis* strain HNA3 were uploaded to the NCBI website and accessed with accession no. CP040881. rRNA sequence of *Alternaria alternata* has accession no. OQ216618, rRNA sequence of *Penicillium expansum* has accession no. OQ201736, rRNA sequence of *Monilinia fructicola* has accession no. OQ216738, rRNA sequence of *Fusarium oxysporum* has accession no. OQ216619, and rRNA sequence of *Cladosporium cladosporioides* has accession no. OQ201835.

## SUPPLEMENTAL MATERIAL

Fig. S1 to Fig. S11 spectrum.00519-23-s0001.pdf Isolation of postharvest fungi, phylogenetic trees, effect of VOCs, IAA, GC chart, pure VOCs.

ASM does not own the copyrights to Supplemental Material that may be linked to, or accessed through, an article. The authors have granted ASM a non-exclusive, world-wide license to publish the Supplemental Material files. Please contact the corresponding author directly for reuse.

## References

[1] Shah A , Nazari M , Antar M , Msimbira LA , Naamala J , Lyu D , Rabileh M , Zajonc J , Smith DL . 2021. PGPR in agriculture: a sustainable approach to increasing climate change resilience. Front Sustain Food Syst 5. doi:10.3389/fsufs.2021.667546

[2] Vågsholm I , Arzoomand NS , Boqvist S . 2020. Food security, safety, and sustainability—getting the trade-offs right. Front Sustain Food Syst 4. doi:10.3389/fsufs.2020.00016

[3] Nicolopoulou-Stamati P , Maipas S , Kotampasi C , Stamatis P , Hens L . 2016. Chemical pesticides and human health: the urgent need for a new concept in agriculture. Front Public Health 4:148. doi:10.3389/fpubh.2016.00148 27486573 PMC4947579

[4] Slavin JL , Lloyd B . 2012. Health benefits of fruits and vegetables. Advances in Nutrition 3:506–516. doi:10.3945/an.112.002154 22797986 PMC3649719

[5] de Oliveira Filho JG , Silva G da C , Cipriano L , Gomes M , Egea MB . 2021. Control of postharvest fungal diseases in fruits using external application of RNAi. J Food Sci 86:3341–3348. doi:10.1111/1750-3841.15816 34272735

[6] Bano A , Gupta A , Prusty MR , Kumar M . 2023. Elicitation of fruit fungi infection and its protective response to improve the Postharvest quality of fruits. Stresses 3:231–255. doi:10.3390/stresses3010018

[7] Liu Y , Galani Yamdeu JH , Gong YY , Orfila C . 2020. A review of postharvest approaches to reduce fungal and mycotoxin contamination of foods. Compr Rev Food Sci Food Saf 19:1521–1560. doi:10.1111/1541-4337.12562 33337083

[8] Nan M , Xue H , Bi Y . 2022. Contamination, detection and control of mycotoxins in fruits and vegetables. Toxins (Basel) 14:309. doi:10.3390/toxins14050309 35622556 PMC9143439

[9] Rosalba Troncoso R , Martín Ernesto Tiznado H . 2014. Chapter 5, *Alternaria Alternata* (black rot, black spot), p 147–187. In Postharvest decay (control strategies). doi:10.1016/B978-0-12-411552-1.00005-3

[10] Vico I , Duduk N , Vasic M , Nikolic M . 2014. Identification of Penicillium expansum causing postharvest blue mold decay of apple fruit. Pesticidi i fitomedicina 29:257–266. doi:10.2298/PIF1404257V

[11] Abdelkhalek A , Behiry SI , Al-Askar AA . 2020. Bacillus velezensis pea1 inhibits Fusarium oxysporum growth and induces systemic resistance to cucumber mosaic virus. Agronomy 10:1312. doi:10.3390/agronomy10091312

[12] Akosah YA , Vologin SG , Lutfullin MT , Hadieva GF , Scyganova NF , Zamalieva FF , Mardanova AM . 2021. Fusarium oxysporum strains from wilting potato plants: potential causal agents of dry rot disease in potato tubers. Res. on Crops 22:49–53. doi:10.31830/2348-7542.2021.012

[13] Ma M , de Silva DD , Taylor PWJ . 2020. Black mould of post-harvest tomato (Solanum lycopersicum) caused by Cladosporium cladosporioides in Australia. Australasian Plant Dis. Notes 15. doi:10.1007/s13314-020-00395-8

[14] Oliveira Lino L , Pacheco I , Mercier V , Faoro F , Bassi D , Bornard I , Quilot-Turion B . 2016. Brown rot strikes prunus fruit: an ancient fight almost always lost. J Agric Food Chem 64:4029–4047. doi:10.1021/acs.jafc.6b00104 27133976

[15] Backer R , Rokem JS , Ilangumaran G , Lamont J , Praslickova D , Ricci E , Subramanian S , Smith DL . 2018. Plant growth-promoting rhizobacteria: context, mechanisms of action, and roadmap to commercialization of biostimulants for sustainable agriculture. Front Plant Sci 871:1473. doi:10.3389/fpls.2018.01473 PMC6206271 30405652

[16] Radhakrishnan R , Hashem A , Abd Allah EF . 2017. Bacillus: a biological tool for crop improvement through bio-molecular changes in adverse environments. Front Physiol 8:667. doi:10.3389/fphys.2017.00667 28932199 PMC5592640

[17] Zaid DS , Cai S , Hu C , Li Z , Li Y . 2022. Comparative genome analysis reveals phylogenetic identity of Bacillus velezensis HNA3 and genomic insights into its plant growth promotion and biocontrol effects. Microbiol Spectr 10:e0216921. doi:10.1128/spectrum.02169-21 35107331 PMC8809340

[18] Miljaković D , Marinković J , Balešević-Tubić S . 2020. The significance of Bacillus spp. In disease suppression and growth promotion of field and vegetable crops. Microorganisms 8:1–19. doi:10.3390/microorganisms8071037 PMC7409232 32668676

[19] Mosela M , Andrade G , Massucato LR , de Araújo Almeida SR , Nogueira AF , de Lima Filho RB , Zeffa DM , Mian S , Higashi AY , Shimizu GD , Teixeira GM , Branco KS , Faria MV , Giacomin RM , Scapim CA , Gonçalves LSA . 2022. Bacillus velezensis strain Ag75 as a new multifunctional agent for biocontrol, phosphate solubilization and growth promotion in maize and soybean crops. Sci Rep 12:15284. doi:10.1038/s41598-022-19515-8 36088482 PMC9464197

[20] Lim JH , Kim SD . 2013. Induction of drought stress resistance by multi-functional PGPR Bacillus licheniformis K11 in pepper. Plant Pathol J 29:201–208. doi:10.5423/PPJ.SI.02.2013.0021 25288947 PMC4174774

[21] Kim SY , Song H , Sang MK , Weon H-Y , Song J . 2017. The complete genome sequence of Bacillus velezensis strain GH1-13 reveals agriculturally beneficial properties and a unique plasmid. J Biotechnol 259:221–227. doi:10.1016/j.jbiotec.2017.06.1206 28690133

[22] Wu Y , Zhou J , Li C , Ma Y . 2019. Antifungal and plant growth promotion activity of volatile organic compounds produced by Bacillus amyloliquefaciens. Microbiologyopen 8:e00813. doi:10.1002/mbo3.813 30907064 PMC6692555

[23] Fincheira P , Parra L , Mutis A , Parada M , Quiroz A . 2017. Volatiles emitted by Bacillus sp. BCT9 act as growth modulating agents on Lactuca sativa seedlings. Microbiol Res 203:47–56. doi:10.1016/j.micres.2017.06.007 28754207

[24] Ghazala I , Chiab N , Saidi MN , Gargouri-Bouzid R . 2022. Volatile organic compounds from Bacillus mojavensis I4 promote plant growth and inhibit phytopathogens. Physiological and Molecular Plant Pathology 121:101887. doi:10.1016/j.pmpp.2022.101887

[25] Arguelles-Arias A , Ongena M , Halimi B , Lara Y , Brans A , Joris B , Fickers P . 2009. Bacillus amyloliquefaciens GA1 as a source of potent antibiotics and other secondary metabolites for biocontrol of plant pathogens. Microb Cell Fact 8:1–12. doi:10.1186/1475-2859-8-63 19941639 PMC2787494

[26] Ramírez V , Munive JA , Cortes L , Muñoz-Rojas J , Portillo R , Baez A . 2020. Long-chain hydrocarbons (C21, C24, and C31) released by Bacillus sp. MH778713 break dormancy of mesquite seeds subjected to chromium stress. Front Microbiol 11:741. doi:10.3389/fmicb.2020.00741 32425908 PMC7212387

[27] Zhou L , Wang J , Wu F , Yin C , Kim KH , Zhang Y . 2022. Termite nest associated Bacillus siamensis YC-9 mediated biocontrol of Fusarium oxysporum f. sp. cucumerinum. Front Microbiol 13:1907. doi:10.3389/fmicb.2022.893393 PMC9198579 35722323

[28] Mateus JR , Dal’Rio I , Jurelevicius D , da Mota FF , Marques JM , Ramos RTJ , da Costa da Silva AL , Gagliardi PR , Seldin L . 2021 Bacillus velezensis t149-19 And Bacillus safensis t052-76 as potential biocontrol agents against foot rot disease in sweet potato. Agriculture (Switzerland) 11:1046. doi:10.3390/agriculture11111046

[29] Xu BH , Ye ZW , Zheng QW , Wei T , Lin JF , Guo LQ . 2018. Isolation and characterization of cyclic lipopeptides with broad-spectrum antimicrobial activity from Bacillus siamensis JFL15. 3 Biotech 8:1–10. doi:10.1007/s13205-018-1443-4 PMC6175734 30333946

[30] Kumar Paul GK , Mahmud S , Dutta AK , Sarkar S , Laboni AA , Hossain M , Nagata A , Karmaker P , Razu MH , Kazi T , Uddin M , Zaman S , Islam MS , Khan M Abu Saleh M n.d. Volatile compounds of Bacillus pseudomycoides induce growth and drought tolerance in wheat (Triticum aestivum L) abbreviations RWC relative water content H 2 O 2 hydrogen peroxide NO nitric oxide CAT catalase SOD superoxide dismutase POD Peroxidases APX Ascorbate peroxidase RMSD root-mean-square deviation. Sci Reports 12:19137. doi:10.1038/s41598-022-22354-2 PMC9646913 36352019

[31] Zhu M , He Y , Li Y , Ren T , Liu H , Huang J , Jiang D , Hsiang T , Zheng L . 2020. Two new biocontrol agents against clubroot caused by Plasmodiophora brassicae. Front Microbiol 10:3099. doi:10.3389/fmicb.2019.03099 32038545 PMC6986203

[32] Cesa-Luna C , Baez A , Quintero-Hernández V , De la Cruz-Enríquez J , Castañeda-Antonio MD , Muñoz-Rojas J . 2020. The importance of antimicrobial compounds produced by beneficial bacteria on the biocontrol of phytopathogens. Acta biol Colomb 25:140–154. doi:10.15446/abc.v25n1.76867

[33] Dheda K , Huggett JF , Bustin SA , Johnson MA , Rook G , Zumla A . 2004. Validation of housekeeping genes for normalizing RNA expression in real-time PCR. Biotechniques 37:112–114. doi:10.2144/04371RR03 15283208

[34] Sibponkrung S , Kondo T , Tanaka K , Tittabutr P , Boonkerd N , Yoshida K , Teaumroong N . 2020. Co-inoculation of Bacillus velezensis strain S141 and Bradyrhizobium strains promotes nodule growth and nitrogen fixation. Microorganisms 8:678. doi:10.3390/microorganisms8050678 32392716 PMC7284691

[35] Odelade KA , Oladeji OS . 2020. Isolation of Phytopathogenic fungi associated with the post-harvest deterioration of watermelon fruits. Scientific African 8:e00366. doi:10.1016/j.sciaf.2020.e00366

[36] Mailafia S , Okoh GR , Olabode HOK , Osanupin R . 2017. Isolation and identification of fungi associated with spoilt fruits vended in Gwagwalada market, Abuja, Nigeria. Vet World 10:393–397. doi:10.14202/vetworld.2017.393-397 28507410 PMC5422242

[37] Qin Q , Fan Y , Jia Q , Duan S , Liu F , Jia B , Wang G , Guo W , Wang C . 2022. The potential of Alternaria toxins production by A. alternata in processing tomatoes. Toxins 14:827. doi:10.3390/toxins14120827 36548724 PMC9781988

[38] Habib W , Masiello M , Chahine-Tsouvalakis H , Al Moussawi Z , Saab C , Tawk ST , Piemontese L , Solfrizzo M , Logrieco AF , Moretti A , Susca A . 2021. Occurrence and characterization of Penicillium species isolated from post-harvest apples in Lebanon. Toxins (Basel) 13:730. doi:10.3390/toxins13100730 34679023 PMC8537683

[39] Perincherry L , Lalak-Kańczugowska J , Stępień Ł . 2019. Fusarium-produced mycotoxins in plant-pathogen interactions. Toxins (Basel) 11:664. doi:10.3390/toxins11110664 31739566 PMC6891594

[40] Saleh I , Al-Thani R . 2019. Fungal food spoilage of supermarkets' displayed fruits. Vet World 12:1877–1883. doi:10.14202/vetworld.2019.1877-1883 32009770 PMC6925035

[41] Papoutsis K , Mathioudakis MM , Hasperué JH , Ziogas V . 2019. Non-chemical treatments for preventing the postharvest fungal rotting of citrus caused by Penicillium digitatum (green mold) and Penicillium italicum (blue mold). Trends in Food Science & Technology 86:479–491. doi:10.1016/j.tifs.2019.02.053

[42] Slathia S , Sharma YP , Hakla HR , Urfan M , Yadav NS , Pal S . 2021. Post-harvest management of alternaria induced rot in tomato fruits with essential oil of Zanthoxylum armatum DC. Front. Sustain. Food Syst 5:225. doi:10.3389/fsufs.2021.679830

[43] Yu L , Qiao N , Zhao J , Zhang H , Tian F , Zhai Q , Chen W . 2020. Postharvest control of Penicillium expansum in fruits: a review. Food Bioscience 36:100633. doi:10.1016/j.fbio.2020.100633

[44] Li H , Yu T . 2001. Effect of chitosan on incidence of brown rot, quality and physiological attributes of postharvest peach fruit. J. Sci. Food Agric 81:269–274. doi:10.1002/1097-0010(20010115)81:2<269::AID-JSFA806>3.0.CO;2-F

[45] Attia MF , Abada KA , Naffa AMAK , Boghdady SF . 2019. Management of potato post harvest tuber rots by some organic acids and essential plant oils. Journal of Phytopathology 47:257–276. doi:10.21608/ejp.2019.123827

[46] Lim SM , Yoon MY , Choi GJ , Choi YH , Jang KS , Shin TS , Park HW , Yu NH , Kim YH , Kim JC . 2017. Diffusible and volatile antifungal compounds produced by an antagonistic Bacillus velezensis G341 against various phytopathogenic fungi. Plant Pathol J 33:488–498. doi:10.5423/PPJ.OA.04.2017.0073 29018312 PMC5624491

[47] Cozzolino ME , Distel JS , García PA , Mascotti ML , Ayub MJ , Benazzi LM , Di Masi SN , Silva PG . 2020. Control of postharvest fungal pathogens in pome fruits by lipopeptides from a Bacillus sp. isolate SL-6. Scientia Horticulturae 261:108957. doi:10.1016/j.scienta.2019.108957

[48] Bayisa RA , Cho JY , Kim KY . 2021. Purification and identification of a new antifungal dipeptide from Bacillus velezensis AR1 culture supernatant. Pest Manag Sci 77:775–779. doi:10.1002/ps.6078 32896082

[49] Cheffi M , Bouket AC , Alenezi FN , Luptakova L , Belka M , Vallat A , Rateb ME , Tounsi S , Triki MA , Belbahri L . 2019. Olea Europaea L. root endophyte Bacillus velezensis OEE1 counteracts oomycete and fungal harmful pathogens and harbours a large repertoire of secreted and volatile metabolites and beneficial functional genes. Microorganisms 7:314. doi:10.3390/microorganisms7090314 31484434 PMC6780883

[50] Toral L , Rodríguez M , Martínez-Checa F , Montaño A , Cortés-Delgado A , Smolinska A , Llamas I , Sampedro I . 2021. Identification of volatile organic compounds in extremophilic bacteria and their effective use in biocontrol of postharvest fungal phytopathogens. Front Microbiol 12:773092. doi:10.3389/fmicb.2021.773092 34867910 PMC8633403

[51] Calvo H , Mendiara I , Arias E , Gracia AP , Blanco D , Venturini ME . 2020. Antifungal activity of the volatile organic compounds produced by Bacillus velezensis strains against postharvest fungal pathogens. Postharvest Biology and Technology 166:111208. doi:10.1016/j.postharvbio.2020.111208

[52] Saeed Q , Xiukang W , Haider FU , Kučerik J , Mumtaz MZ , Holatko J , Naseem M , Kintl A , Ejaz M , Naveed M , Brtnicky M , Mustafa A . 2021. Rhizosphere bacteria in plant growth promotion, biocontrol, and bioremediation of contaminated sites: a comprehensive review of effects and mechanisms. Int J Mol Sci 22:10529. doi:10.3390/ijms221910529 34638870 PMC8509026

[53] Vishwakarma K , Kumar N , Shandilya C , Mohapatra S , Bhayana S , Varma A . 2020. Revisiting plant-microbe interactions and microbial consortia application for enhancing sustainable agriculture: a review. Front Microbiol 11:560406. doi:10.3389/fmicb.2020.560406 33408698 PMC7779480

[54] Thomas G , Withall D , Birkett M . 2020. Harnessing microbial volatiles to replace pesticides and fertilizers. Microb Biotechnol 13:1366–1376. doi:10.1111/1751-7915.13645 32767638 PMC7415372

[55] Rabbee MF , Ali MS , Choi J , Hwang BS , Jeong SC , Baek K-H . 2019. Bacillus velezensis: a valuable member of bioactive molecules within plant microbiomes. Molecules 24:1046. doi:10.3390/molecules24061046 30884857 PMC6470737

[56] Kondo T , Sibponkrung S , Hironao KY , Tittabutr P , Boonkerd N , Ishikawa S , Ashida H , Teaumroong N , Yoshida KI . 2023. Bacillus velezensis S141, a soybean growth-promoting bacterium, hydrolyzes isoflavone glycosides into aglycones. J Gen Appl Microbiol. doi:10.2323/jgam.2023.02.002 36858546

[57] Teixeira GM , Mosela M , Nicoletto MLA , Ribeiro RA , Hungria M , Youssef K , Higashi AY , Mian S , Ferreira AS , Gonçalves LSA , Pereira U de P , de Oliveira AG . 2020. Genomic insights into the antifungal activity and plant growth-promoting ability in Bacillus velezensis CMRP 4490. Front Microbiol 11:618415. doi:10.3389/fmicb.2020.618415 33519779 PMC7844144

[58] Olanrewaju OS , Glick BR , Babalola OO . 2017. Mechanisms of action of plant growth promoting bacteria. World J Microbiol Biotechnol 33:197. doi:10.1007/s11274-017-2364-9 28986676 PMC5686270

[59] Tahir HAS , Gu Q , Wu H , Raza W , Hanif A , Wu L , Colman MV , Gao X . 2017. Plant growth promotion by volatile organic compounds produced by Bacillus subtilis SYST2. Front Microbiol 8:171. doi:10.3389/fmicb.2017.00171 28223976 PMC5293759

[60] Bavaresco LG , Osco LP , Araujo ASF , Mendes LW , Bonifacio A , Araújo FF . 2020. Bacillus subtilis can modulate the growth and root architecture in soybean through volatile organic compounds. Theor. Exp. Plant Physiol 32:99–108. doi:10.1007/s40626-020-00173-y

[61] Fincheira P , Quiroz A . 2018. Microbial volatiles as plant growth inducers. Microbiol Res 208:63–75. doi:10.1016/j.micres.2018.01.002 29551213

[62] Fincheira P , Quiroz A , Tortella G , Diez MC , Rubilar O . 2021. Current advances in plant-microbe communication via volatile organic compounds as an innovative strategy to improve plant growth. Microbiol Res 247:126726. doi:10.1016/j.micres.2021.126726 33640574

[63] Sharifi R , Ryu CM . 2018. Revisiting bacterial volatile-mediated plant growth promotion: lessons from the past and objectives for the future. Ann Bot 122:349–358. doi:10.1093/aob/mcy108 29982345 PMC6110341

[64] Raza W , Wei Z , Ling N , Huang Q , Shen Q . 2016. Effect of organic fertilizers prepared from organic waste materials on the production of antibacterial volatile organic compounds by two biocontrol bacillus amyloliquefaciens strains. J Biotechnol 227:43–53. doi:10.1016/j.jbiotec.2016.04.014 27067079

[65] Gotor-Vila A , Teixidó N , Di Francesco A , Usall J , Ugolini L , Torres R , Mari M . 2017. Antifungal effect of volatile organic compounds produced by Bacillus amyloliquefaciens CPA-8 against fruit pathogen decays of cherry. Food Microbiol 64:219–225. doi:10.1016/j.fm.2017.01.006 28213029

[66] Raza W , Wang J , Wu Y , Ling N , Wei Z , Huang Q , Shen Q . 2016. Effects of volatile organic compounds produced by Bacillus amyloliquefaciens on the growth and virulence traits of tomato bacterial wilt pathogen Ralstonia solanacearum. Appl Microbiol Biotechnol 100:7639–7650. doi:10.1007/s00253-016-7584-7 27183998

[67] Fiddaman PJ , Rossall S . 1994. Effect of substrate on the production of antifungal Volatiles from Bacillus subtilis. J Appl Bacteriol 76:395–405. doi:10.1111/j.1365-2672.1994.tb01646.x 8200865

[68] Saleh AE , Ul-Hassan Z , Zeidan R , Al-Shamary N , Al-Yafei T , Alnaimi H , Higazy NS , Migheli Q , Jaoua S . 2021. Biocontrol activity of Bacillus Megaterium BM344-1 against Toxigenic fungi. ACS Omega 6:10984–10990. doi:10.1021/acsomega.1c00816 34056251 PMC8153935

[69] Li M , Guo R , Yu F , Chen X , Zhao H , Li H , Wu J . 2018. Indole-3-acetic acid biosynthesis pathways in the plant-beneficial bacterium arthrobacter pascens ZZ21. Int J Mol Sci 19:443. doi:10.3390/ijms19020443 29389906 PMC5855665

[70] Abdel-Hamid MS , Fouda A , El-Ela HKA , El-Ghamry AA , Hassan SED . 2021. Plant growth-promoting properties of bacterial endophytes isolated from roots of Thymus vulgaris L. and investigate their role as biofertilizers to enhance the essential oil contents. Biomol Concepts 12:175–196. doi:10.1515/bmc-2021-0019 35041305

[71] Idris ESE , Iglesias DJ , Talon M , Borriss R . 2007. Tryptophan-dependent production of Indole-3-acetic acid (IAA) affects level of plant growth promotion by Bacillus amyloliquefaciens FZB42. Mol Plant Microbe Interact 20:619–626. doi:10.1094/MPMI-20-6-0619 17555270

[72] Liu WH , Chen FF , Wang CE , Fu HH , Fang XQ , Ye JR , Shi JY . 2019. Indole-3-acetic acid in Burkholderia pyrrocinia JK-SH007: enzymatic identification of the Indole-3-Acetamide synthesis pathway. Front Microbiol 10:2559. doi:10.3389/fmicb.2019.02559 31749788 PMC6848275

[73] Zhang P , Jin T , Kumar Sahu S , Xu J , Shi Q , Liu H , Wang Y . 2019. The distribution of Tryptophan-dependent Indole-3-acetic acid synthesis pathways in bacteria unraveled by large-scale genomic analysis. Molecules 24:1411. doi:10.3390/molecules24071411 30974826 PMC6479905

[74] David E , Niculescu VC . 2021. Volatile organic compounds (VOCs) as environmental pollutants: occurrence and mitigation using nanomaterials. Int J Environ Res Public Health 18:13147. doi:10.3390/ijerph182413147 34948756 PMC8700805

[75] Ueta I , Takenaka R , Fujimura K , Yoshimura T , Narukami S , Mochizuki S , Maeda T . 2020. Simultaneous extraction and determination of volatile organic compounds and semi-volatile organic compounds in indoor air using multi-bed solid phase extraction device. Anal Sci 36:1071–1074. doi:10.2116/analsci.20P022 32307348

[76] Gao Z , Zhang B , Liu H , Han J , Zhang Y . 2017. Identification of endophytic Bacillus velezensis ZSY-1 strain and antifungal activity of its volatile compounds against Alternaria solani and Botrytis cinerea. Biological Control 105:27–39. doi:10.1016/j.biocontrol.2016.11.007

[77] He Y , Peng J , Jia N , Wang X , Ma J , Wang H , Zhang C , Wang E , Hu D , Wang Z . 2023. Bacillus vlezensis Wsw007 different concentrations volatile organic compounds stimulated tobacco growth by up-regulating the expression of genes related to plant growth and development. doi:10.21203/rs.3.rs-2871463/v1 PMC9164152 35668752

[78] Hu J , Dong B , Wang D , Meng H , Li X , Zhou H . 2022. Genomic and metabolic features of Bacillus cereus, inhibiting the growth of Sclerotinia sclerotiorum by synthesizing secondary metabolites. Arch Microbiol 205:8. doi:10.1007/s00203-022-03351-5 36454319 PMC9715469

[79] Mohamad OAA , Li L , Ma J-B , Hatab S , Xu L , Guo J-W , Rasulov BA , Liu Y-H , Hedlund BP , Li WJ . 2018. Evaluation of the antimicrobial activity of endophytic bacterial populations from Chinese traditional medicinal plant licorice and characterization of the bioactive secondary metabolites produced by Bacillus Atrophaeus against Verticillium dahliae. Front Microbiol 9:924. doi:10.3389/fmicb.2018.00924 29867835 PMC5954123

[80] Kashyap AS , Manzar N , Nebapure SM , Rajawat MVS , Deo MM , Singh JP , Kesharwani AK , Singh RP , Dubey SC , Singh D . 2022. Unraveling microbial volatile elicitors using a transparent methodology for induction of systemic resistance and regulation of antioxidant genes at expression levels in chili against bacterial wilt disease. Antioxidants 11:404. doi:10.3390/antiox11020404 35204287 PMC8869530

[81] Selvamani K , Tadigiri S , Das D . 2020. Isolation and characterization of chemical constituents from B. Amyloliquefaciens and their Nematicidal activity. Article in journal of entomology and zoology studies 8:2062–2066. https://api.semanticscholar.org/CorpusID:222260468.

[82] Keerthana S , Rajeswari E , Jayamani P . 2018. Exploiting biocontrol potential of Bacillus amyloliquefaciens (sic.) fukumoto for the management of mungbean anthracnose. Madras Agricultural Journal 105. doi:10.29321/MAJ.2018.000149

[83] Chen J , Hu L , Chen N , Jia R , Ma Q , Wang Y . 2021. The biocontrol and plant growth-promoting properties of Streptomyces alfalfae XN-04 revealed by functional and genomic analysis. Front Microbiol 12:745766. doi:10.3389/fmicb.2021.745766 34630371 PMC8493286

[84] Pradhan PC , Mukhopadhyay A , Kumar R , Kundu A , Patanjali N , Dutta A , Kamil D , Bag TK , Aggarwal R , Bharadwaj C , Singh PK , Singh A . 2022. Performance appraisal of Trichoderma viride based novel tablet and powder formulations for management of Fusarium wilt disease in chickpea. Front Plant Sci 13:3645. doi:10.3389/fpls.2022.990392 PMC9585344 36275506

[85] Pinto MEA , Araújo SG , Morais MI , Sá NP , Lima CM , Rosa CA , Siqueira EP , Johann S , Lima LARS . 2017. Antifungal and antioxidant activity of fatty acid methyl esters from vegetable oils. An Acad Bras Cienc 89:1671–1681. doi:10.1590/0001-3765201720160908 28876392

[86] Rajaofera MJN , Wang Y , Dahar GY , Jin P , Fan L , Xu L , Liu W , Miao W . 2019. Volatile organic compounds of Bacillus atrophaeus HAB-5 inhibit the growth of Colletotrichum gloeosporioides. Pestic Biochem Physiol 156:170–176. doi:10.1016/j.pestbp.2019.02.019 31027577

[87] Li X , Wang X , Shi X , Wang B , Li M , Wang Q , Zhang S . 2020. Antifungal effect of volatile organic compounds from Bacillus velezensis CT32 against Verticillium dahliae and Fusarium oxysporum. Processes 8:1674. doi:10.3390/pr8121674

[88] Thibane VS , Ells R , Hugo A , Albertyn J , van Rensburg WJJ , Van Wyk PWJ , Kock JLF , Pohl CH . 2012. Polyunsaturated fatty acids cause apoptosis in C. albicans and C. dubliniensis biofilms. Biochim Biophys Acta 1820:1463–1468. doi:10.1016/j.bbagen.2012.05.004 22609876

[89] Suzuki K , Shono F , Kai H , Uno T , Uyeda M . 2010. Inhibition of topoisomerases by fatty acids. J Enzyme Inhib 15:357–366. doi:10.1080/14756360009040693 10995067

[90] Corsetti A , Gobbetti M , Rossi J , Damiani P . 1998. Antimould activity of sourdough lactic acid bacteria: identification of a mixture of organic acids produced by Lactobacillus sanfrancisco CB1. Appl Microbiol Biotechnol 50:253–256. doi:10.1007/s002530051285 9763693

[91] Wang Z , Zhong T , Chen X , Yang B , Du M , Wang K , Zalán Z , Kan J . 2021. Potential of volatile organic compounds emitted by pseudomonas fluorescens ZX as biological fumigants to control citrus green mold decay at postharvest. J Agric Food Chem 69:2087–2098. doi:10.1021/acs.jafc.0c07375 33560120

[92] Ling L , Luo H , Yang C , Wang Y , Cheng W , Pang M , Jiang K . 2022. Volatile organic compounds produced by Bacillus velezensis L1 as a potential biocontrol agent against postharvest diseases of wolfberry. Front Microbiol 13:987844. doi:10.3389/fmicb.2022.987844 36090114 PMC9449519

[93] Lee J-H , Lee J . 2010. Indole as an intercellular signal in microbial communities. FEMS Microbiol Rev 34:426–444. doi:10.1111/j.1574-6976.2009.00204.x 20070374

[94] Bailly A , Groenhagen U , Schulz S , Geisler M , Eberl L , Weisskopf L . 2014. The inter-kingdom volatile signal Indole promotes root development by interfering with auxin signalling. Plant J 80:758–771. doi:10.1111/tpj.12666 25227998

[95] Pelagio-Flores R , Ortíz-Castro R , Méndez-Bravo A , Macías-Rodríguez L , López-Bucio J . 2011. Serotonin, a tryptophan-derived signal conserved in plants and animals, regulates root system architecture probably acting as a natural auxin inhibitor in Arabidopsis thaliana. Plant Cell Physiol 52:490–508. doi:10.1093/pcp/pcr006 21252298

[96] Jiao J , Ma Y , Chen S , Liu C , Song Y , Qin Y , Yuan C , Liu Y . 2016. Melatonin-producing endophytic bacteria from grapevine roots promote the abiotic stress-induced production of endogenous melatonin in their hosts. Front Plant Sci 7:1387. doi:10.3389/fpls.2016.01387 27708652 PMC5030213

[97] Ma Y , Jiao J , Fan X , Sun H , Zhang Y , Jiang J , Liu C . 2016. Endophytic bacterium Pseudomonas fluorescens RG11 may transform tryptophan to melatonin and promote endogenous melatonin levels in the roots of four grape cultivars. Front Plant Sci 7:2068. doi:10.3389/fpls.2016.02068 28119731 PMC5223058

[98] Wang WY , Kong WL , Liao YCZ , Zhu LH . 2022. Identification of Bacillus velezensis SBB and its antifungal effects against Verticillium dahliae. JoF 8:1021. doi:10.3390/jof8101021 36294586 PMC9604920

[99] Reva ON , Swanevelder DZH , Mwita LA , Mwakilili AD , Muzondiwa D , Joubert M , Chan WY , Lutz S , Ahrens CH , Avdeeva LV , Kharkhota MA , Tibuhwa D , Lyantagaye S , Vater J , Borriss R , Meijer J . 2019. Genetic, epigenetic and phenotypic diversity of four Bacillus velezensis strains used for plant protection or as probiotics. Front Microbiol 10:2610. doi:10.3389/fmicb.2019.02610 31803155 PMC6873887

[100] Niemhom N , Kittiwongwattana C . 2022. Biocontrol potential, genome and nonribosomal peptide synthetase gene expression of Bacillus velezensis 2211. Curr Appl Sci Technol 23. doi:10.55003/cast.2022.03.23.005

[101] Xu L , Wang L , Chen L , Xie F , Li Y . 2013. Identification of Bacillus strain Hna3 and its bioactive compounds with a broad spectrum of antifungal activity. Journal of Huazhong Agricultural University 32:21–27. http://hnxbl.cnjournals.net/hznydxzr/article/abstract/20130304.

[102] Alsohaili SA , Bani-Hasan BM . 2018. Morphological and molecular identification of fungi isolated from different environmental sources in the northern Eastern desert of Jordan. Jordan J Biol Sci 11. https://jjbs.hu.edu.jo/files/v11n3/Binder11n3.pdf.

[103] Abdullah Q , Mahmoud A , Al-Harethi A . 2016. Isolation and identification of fungal post-harvest rot of some fruits in Yemen. PSM Microbiol 01:36–44. https://api.semanticscholar.org/CorpusID:56372722.

[104] Al-Jaradi A , Al-Mahmooli I , Janke R , Maharachchikumbura S , Al-Saady N , Al-Sadi AM . 2018. Isolation and identification of pathogenic fungi and oomycetes associated with beans and cowpea root diseases in Oman. PeerJ 6:e6064. doi:10.7717/peerj.6064 30581667 PMC6295327

[105] Kimura M . 1980. A simple method for estimating evolutionary rates of base substitutions through comparative studies of nucleotide sequences. J Mol Evol 16:111–120. doi:10.1007/BF01731581 7463489

[106] Aoudou Y , Phalone MEG . 2016. Isolation and pathogenicity evaluation of postharvest fungal of some fruits in Cameroon. IJEAB 2:56–60. doi:10.22161/ijeab/2.1.9

[107] Yu Z , Han C , Yu B , Zhao J , Yan Y , Huang S , Liu C , Xiang W . 2020. Taxonomic characterization, and secondary metabolite analysis of Streptomyces triticiradicis sp. nov.: a novel actinomycete with antifungal activity. Microorganisms 8:77. doi:10.3390/microorganisms8010077 31948045 PMC7023189

[108] Rouissi W , Ugolini L , Martini C , Lazzeri L , Mari M . 2013. Control of postharvest fungal pathogens by antifungal compounds from Penicillium expansum. J Food Prot 76:1879–1886. doi:10.4315/0362-028X.JFP-13-072 24215691

[109] Li MW , Wang Z , Jiang B , Kaga A , Wong FL , Zhang G , Han T , Chung G , Nguyen H , Lam HM . 2020. Impacts of genomic research on soybean improvement in East Asia. Theor Appl Genet 133:1655–1678. doi:10.1007/s00122-019-03462-6 31646364 PMC7214498

[110] Ku Y , Xu G , Tian X , Xie H , Yang X , Cao C , Chen Y . 2018. Correction: root colonization and growth promotion of soybean, wheat and Chinese cabbage by Bacillus cereus YL6. PLoS One 13:e0210035. doi:10.1371/journal.pone.0210035 30462642 PMC6248894

[111] Tahir HAS , Gu Q , Wu H , Raza W , Safdar A , Huang Z , Rajer FU , Gao X . 2017. Effect of volatile compounds produced by Ralstonia solanacearum on plant growth promoting and systemic resistance inducing potential of Bacillus volatiles. BMC Plant Biol 17:133. doi:10.1186/s12870-017-1083-6 28768498 PMC5541429

[112] Sharma A , Shukla A , Gupta M . 2023. Effect of bioagents on cucumber seed mycoflora, seed germination, and seedling vigour. Sci Rep 13:6052. doi:10.1038/s41598-023-30253-3 37055421 PMC10101947

[113] Park YS , Dutta S , Ann M , Raaijmakers JM , Park K . 2015. Promotion of plant growth by Pseudomonas fluorescens strain SS101 via novel volatile organic compounds. Biochem Biophys Res Commun 461:361–365. doi:10.1016/j.bbrc.2015.04.039 25892516

[114] Timm CM , Lloyd EP , Egan A , Mariner R , Karig D . 2018. Direct growth of bacteria in headspace vials allows for screening of volatiles by gas chromatography mass spectrometry. Front Microbiol 9:491. doi:10.3389/fmicb.2018.00491 29662472 PMC5890184

[115] Lei C , Teng Y , He L , Sayed M , Mu J , Xu F , Zhang X , Kumar A , Sundaram K , Sriwastva MK , Zhang L , Chen SY , Feng W , Zhang S , Yan J , Park JW , Merchant ML , Zhang X , Zhang HG . 2021. Lemon exosome-like nanoparticles enhance stress survival of gut bacteria by RNase P-mediated specific tRNA decay. iScience 24:102511. doi:10.1016/j.isci.2021.102511 34142028 PMC8188359

