# Supplementary material for: Identification of bioactive compounds of Bacillus velezensis HNA3 that contribute to its dual effects as plant growth promoter and biocontrol against post-harvested fungi
Source: Microbiol Spectr. 2023 Oct 9;11(6):e00519-23. doi: 10.1128/spectrum.00519-23 (PMC10715170; doi:10.1128/spectrum.00519-23)
Supplement: Fig. S1 to Fig. S11 — Isolation of postharvest fungi, phylogenetic trees, effect of VOCs, IAA, GC chart, pure VOCs. [file spectrum.00519-23-s0001.pdf]

Figure S1.

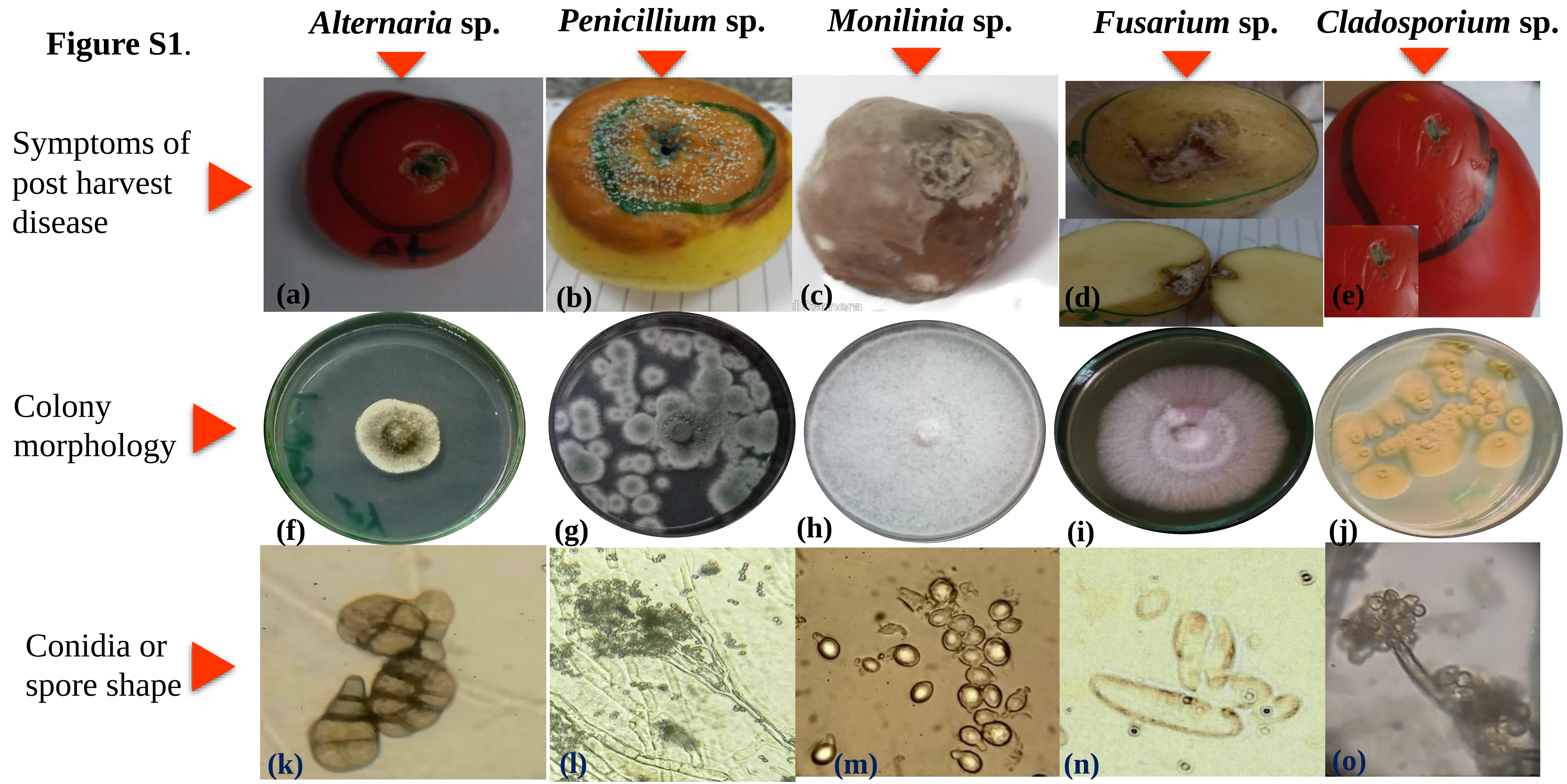

Fig. S1: Illustrates the five isolated postharvest fungi: (a-e) symptoms of the postharvest disease on fruit and vegetable, (f-j) Colony morphology of postharvest fungi grown on potato dextrose agar (PDA), and (k–o) microscopic identification of conidia or spore shape. (a, f, and k identified as *Alternaria* sp.), (b, g, and l identified as *penicillium* sp.), (c, h, and m identified as *Monilinia* sp.), (d, i, and n identified as *Fusarium* sp.), and (e, j, and o identified as *Cladosporium* sp.)

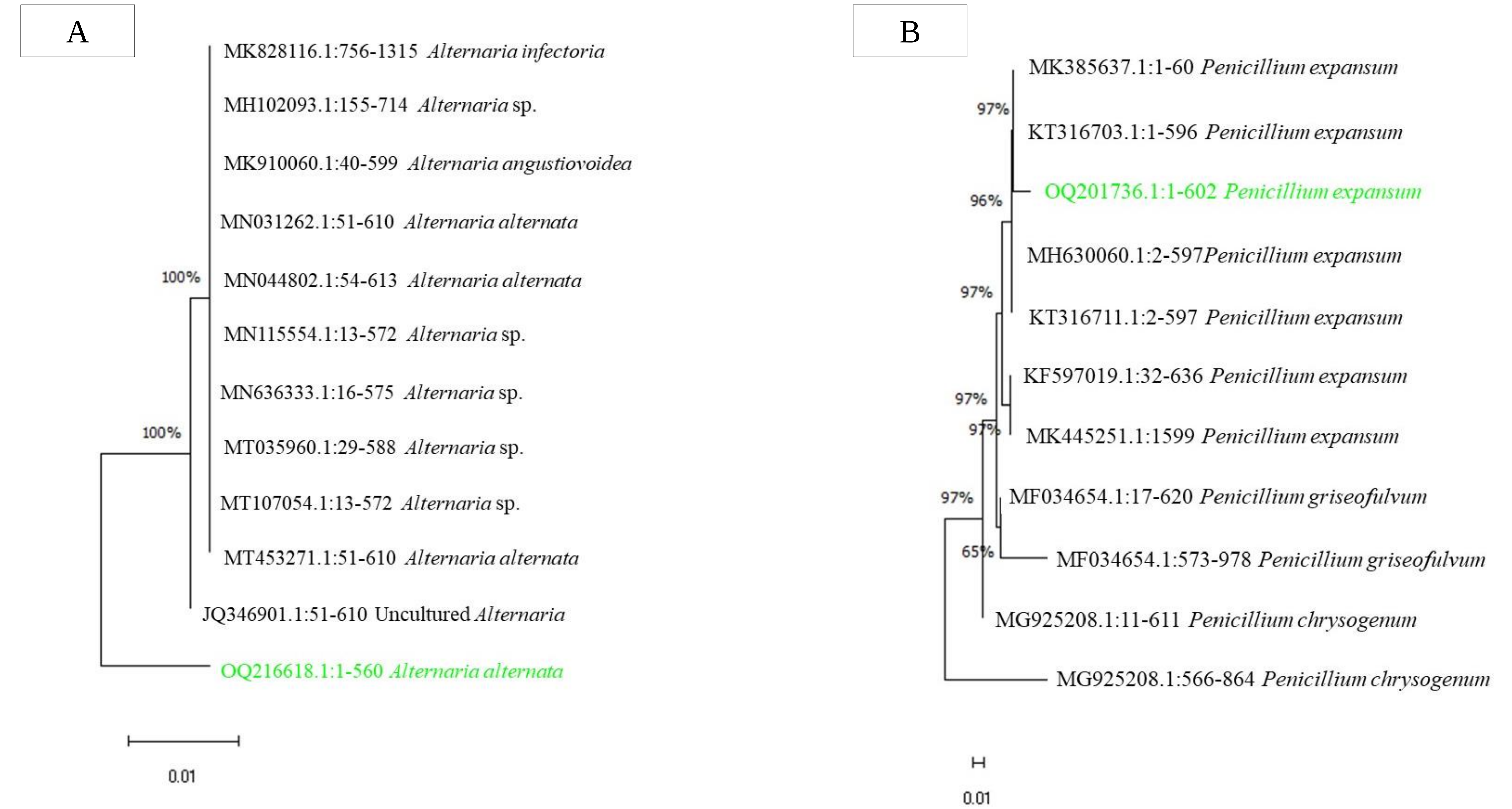

Fig. S2: The maximum likelihood (ML) tree based on the ITS region reveals the associations of isolated fungal strains compared to sequences derived from the NCBI database. Numbers on branches indicate bootstrap values greater than 50% in 1000 replicates. (**A**) *Alternaria alternata* and *(***B**) *Penicillium expansum.*

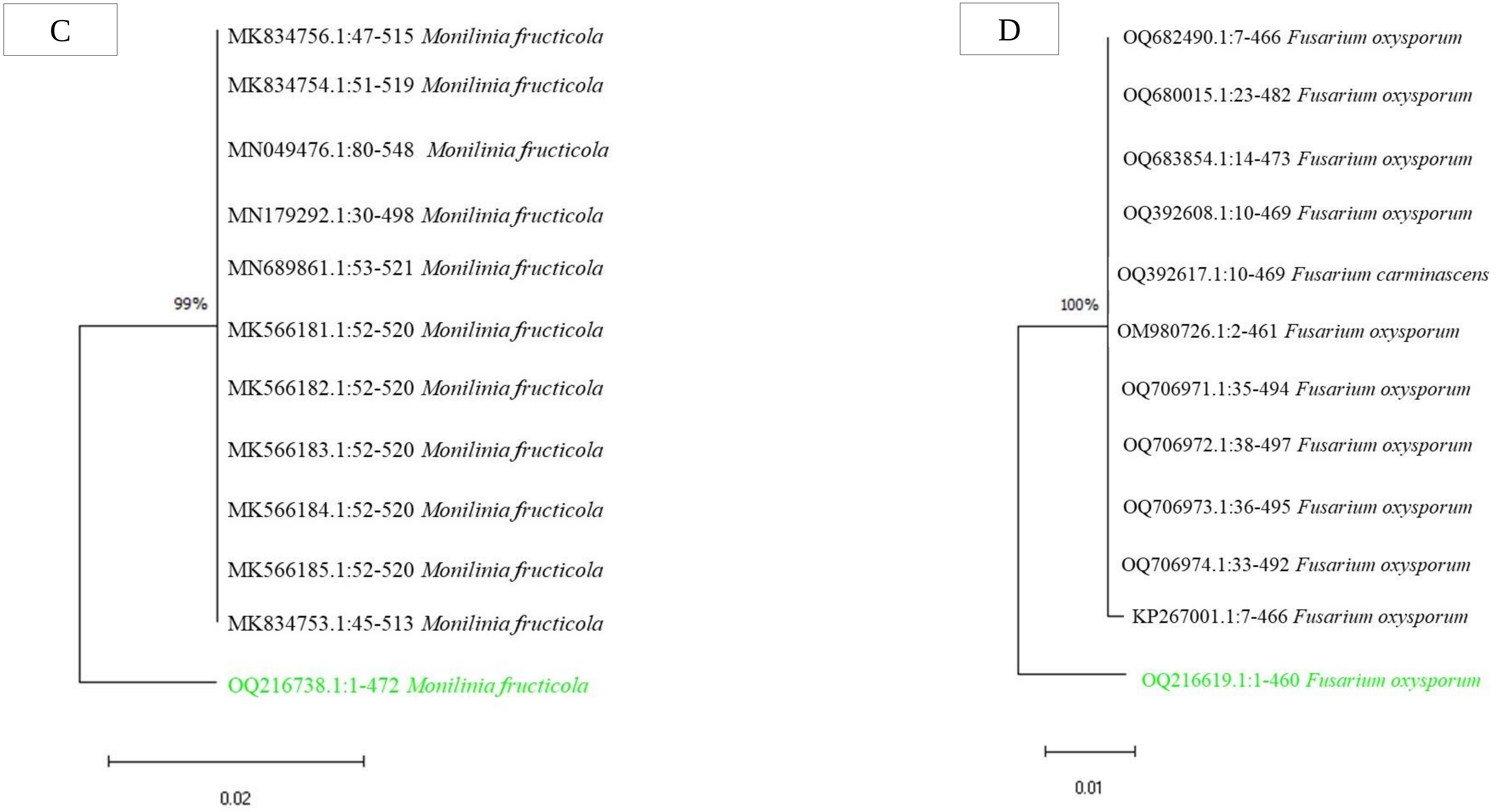

Fig. S3: The maximum likelihood (ML) tree based on the ITS region reveals the associations of isolated fungal strains compared to sequences derived from the NCBI database. Numbers on branches indicate bootstrap values greater than 50% in 1000 replicates. (**C**) *Monilinia fructicola* and **(D)** *Fusarium oxysporum.*

E

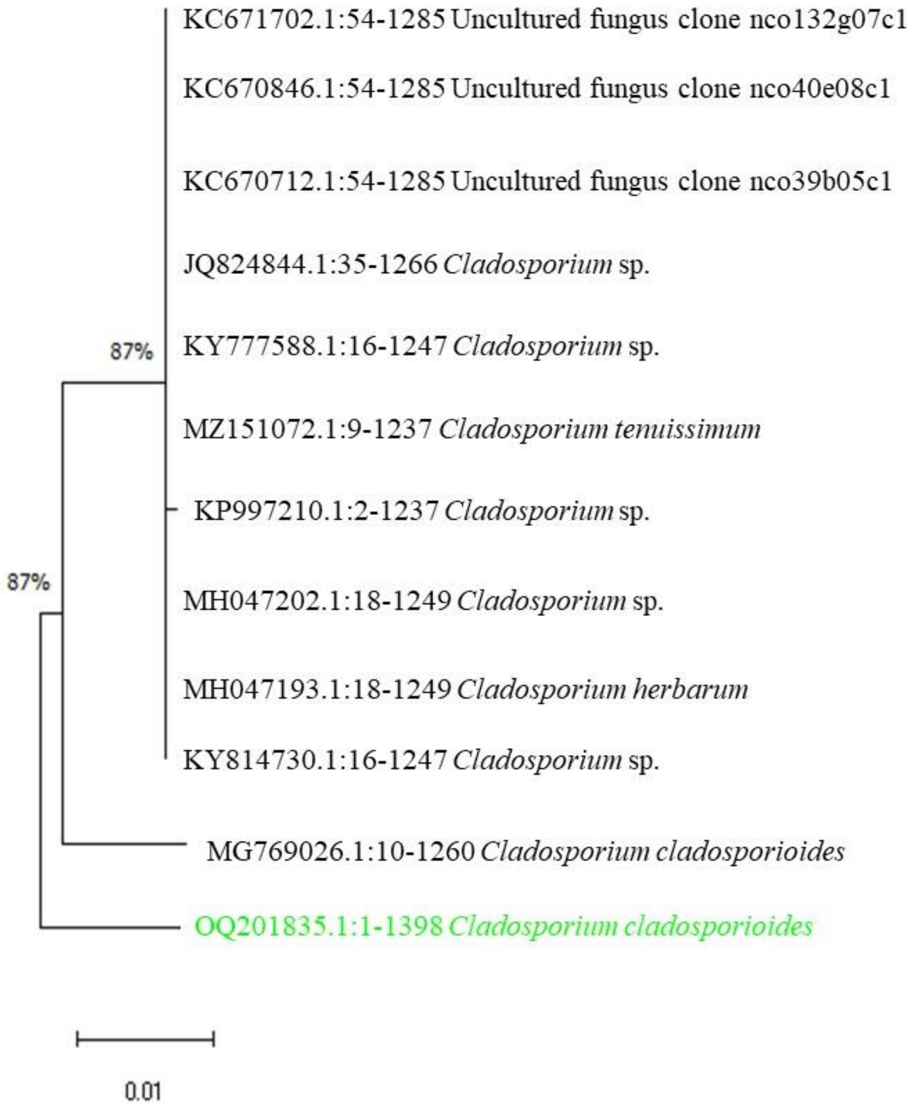

Fig. S4: The maximum likelihood (ML) tree based on the 18 sRNA sequence reveals the associations of isolated fungal strains compared to sequences derived from the NCBI database. Numbers on branches indicate bootstrap values greater than 50% in 1000 replicates. (**E**) *Cladosporium cladosporioides.*

Figure S5

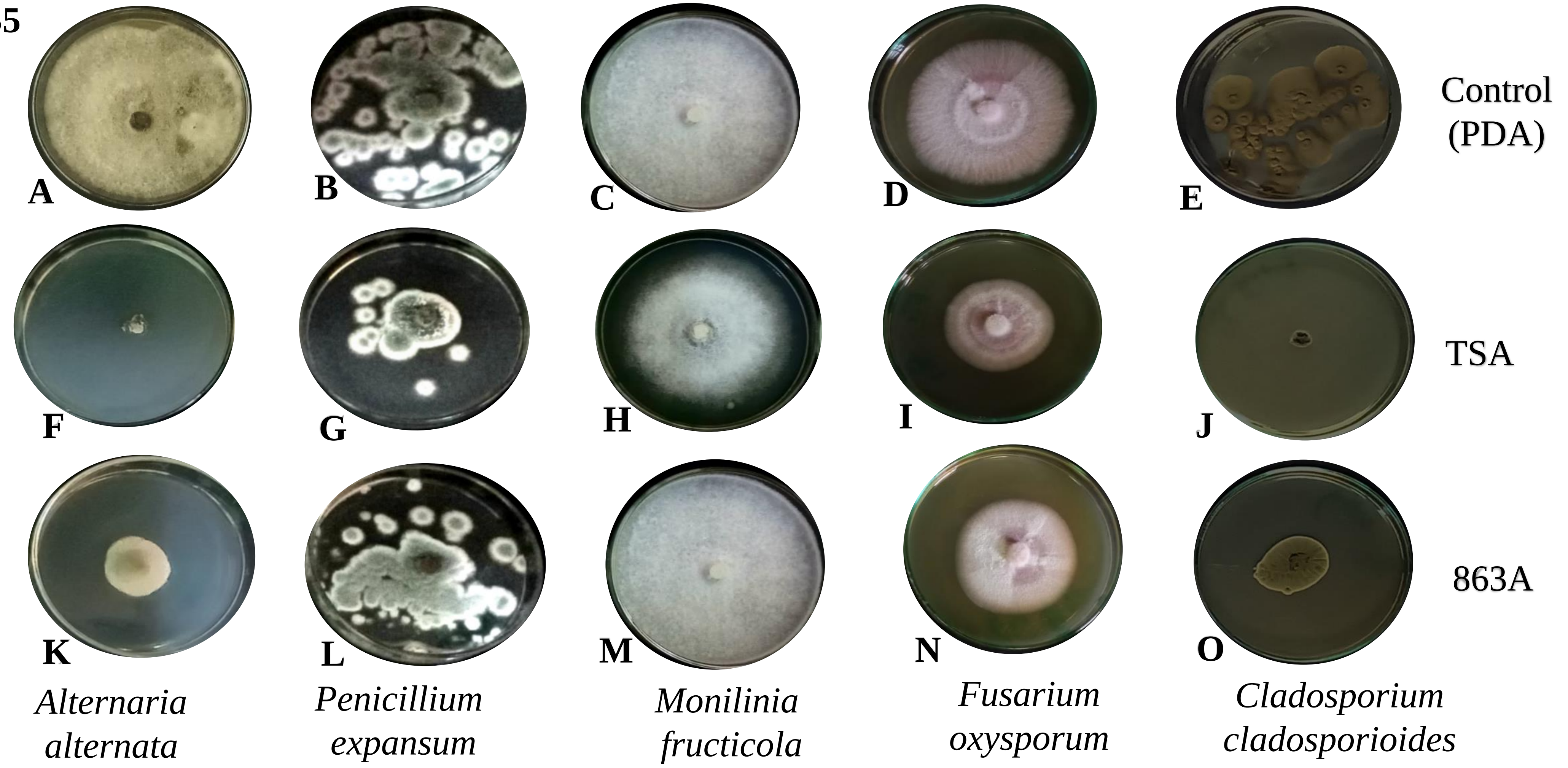

Fig. S5: Antifungal activity of VOCs emitted from *Bacillus velezensis* HNA3 against postharvest pathogenic fungi, using two growth media (TSA and 863 A) for HNA3 growth, while PDA medium was used for fungal growth (Control). (A, F, and K) *Alternaria alternata*, (B, G, and L) *Penicillium expansum*, (C, H, and M) *Monilinia fructicola*, (D, I, and N) *Fusarium oxysporum*, and (E, J, and O) *Cladosporium cladosporioides*.

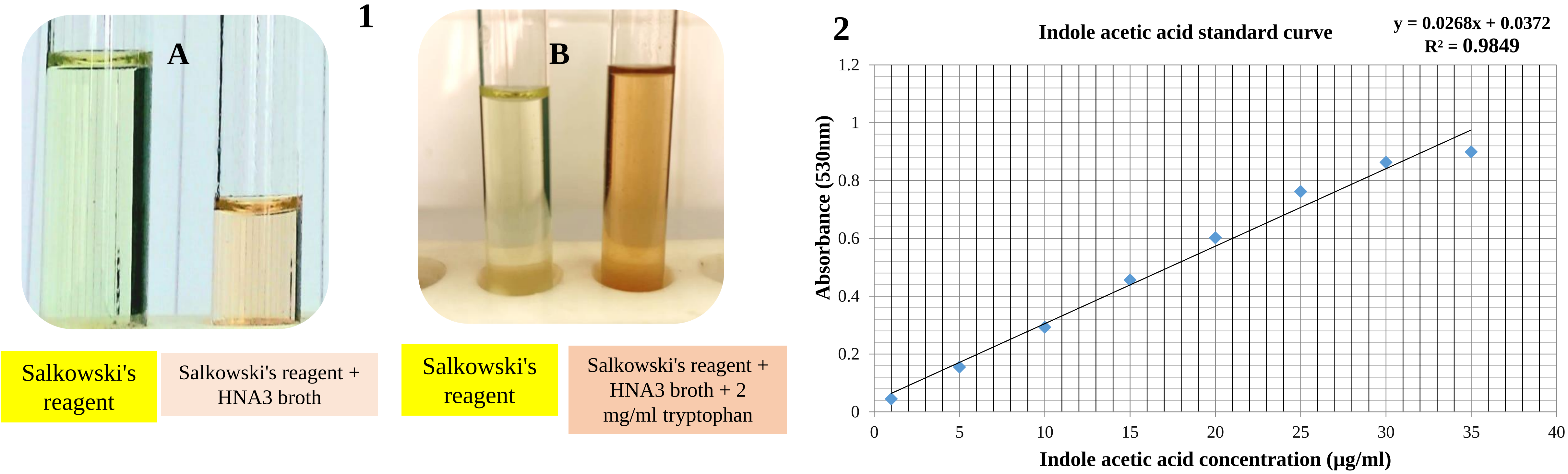

Fig. S6: (1) Colorimetric assay using Salkowski's reagent to measure indole acetic acid (IAA) concentration produced by HNA3, (**A**) the color intensity of the reaction of HNA3 broth + Salkowski's reagent in the absence of tryptophan and (**B**) color intensity of the reaction of HNA3 broth + Salkowski's reagent + 2 mg/ml of tryptophan after 10 days of incubation in dark at 37°C . **(2)** Indole acetic acid standard curve, Absorbance value of IAA different concentrations (1, 5, 15, 20, 25, 30, and 35 µg/ml) have been measured at 530 nm and equation calculated as $y = 0.0268x + 0.0372$.

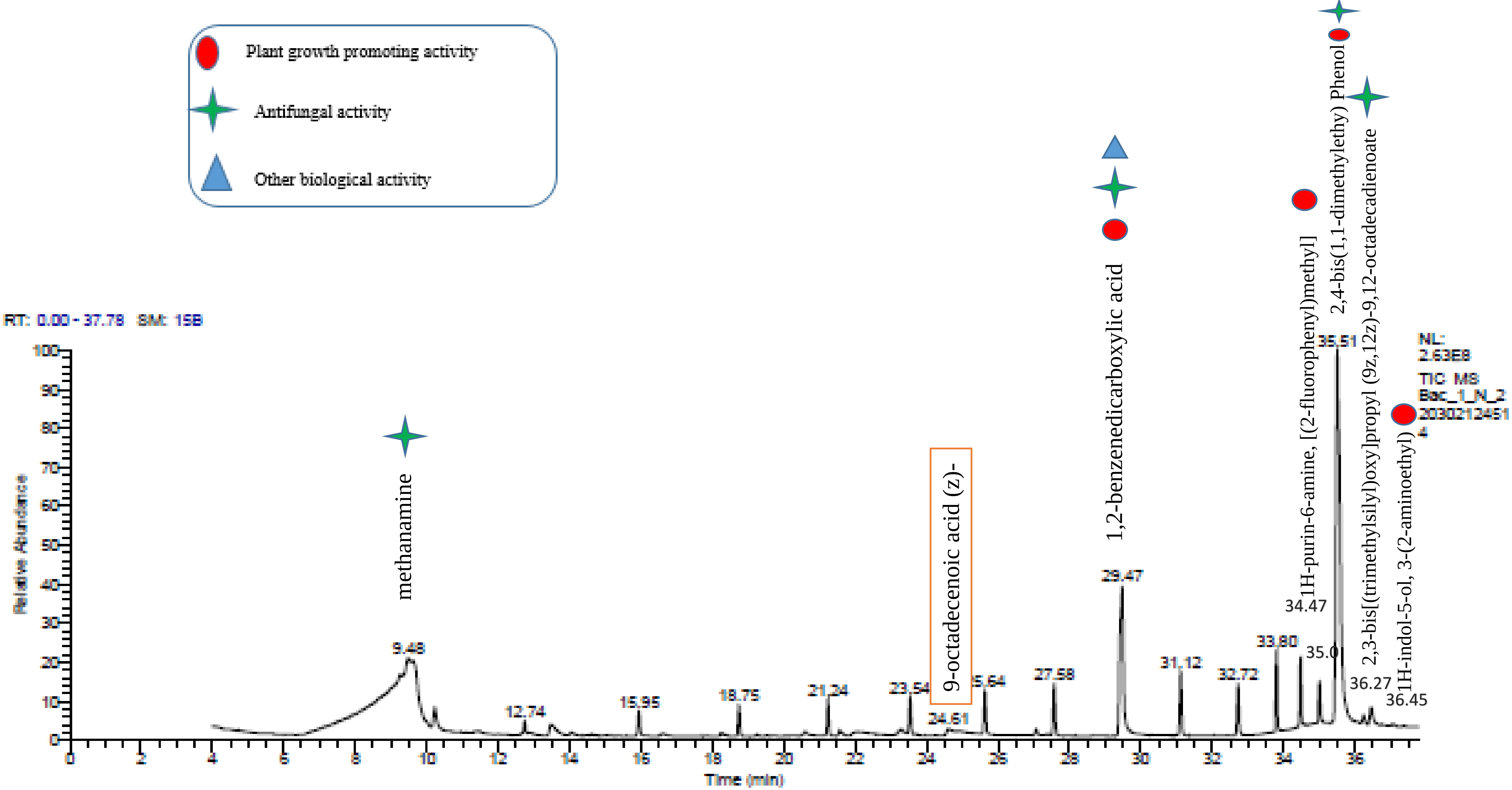

Fig. S7: Displays the GC-mass chart for semi-volatile active compounds in the extract of HNA3. The compound names are indicated above each peak, with 9-octadecenoic acid (z)- being present in both HNA3 samples and the control sample. Different shapes (circle, triangle, and star) are used to signify the known biological functions of the compounds.

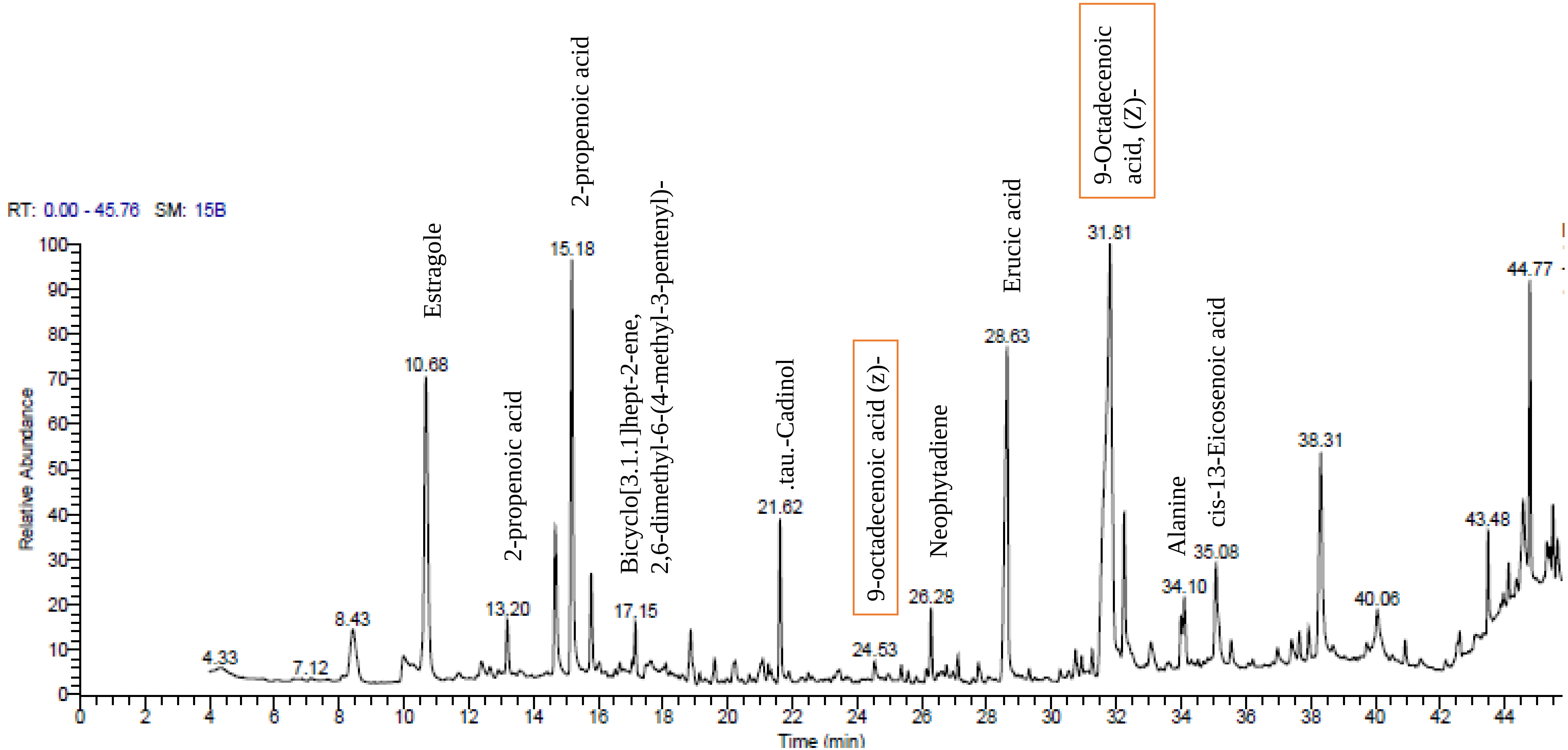

Fig. S8: GC-mass chart for semi-volatiles in the extract of TSB medium without bacteria (control). Identified compounds are indicated above their respective peaks. Notably, 9-octadecenoic acid (z)-, highlighted within a red box, was detected in both HNA3 samples and the control samples.

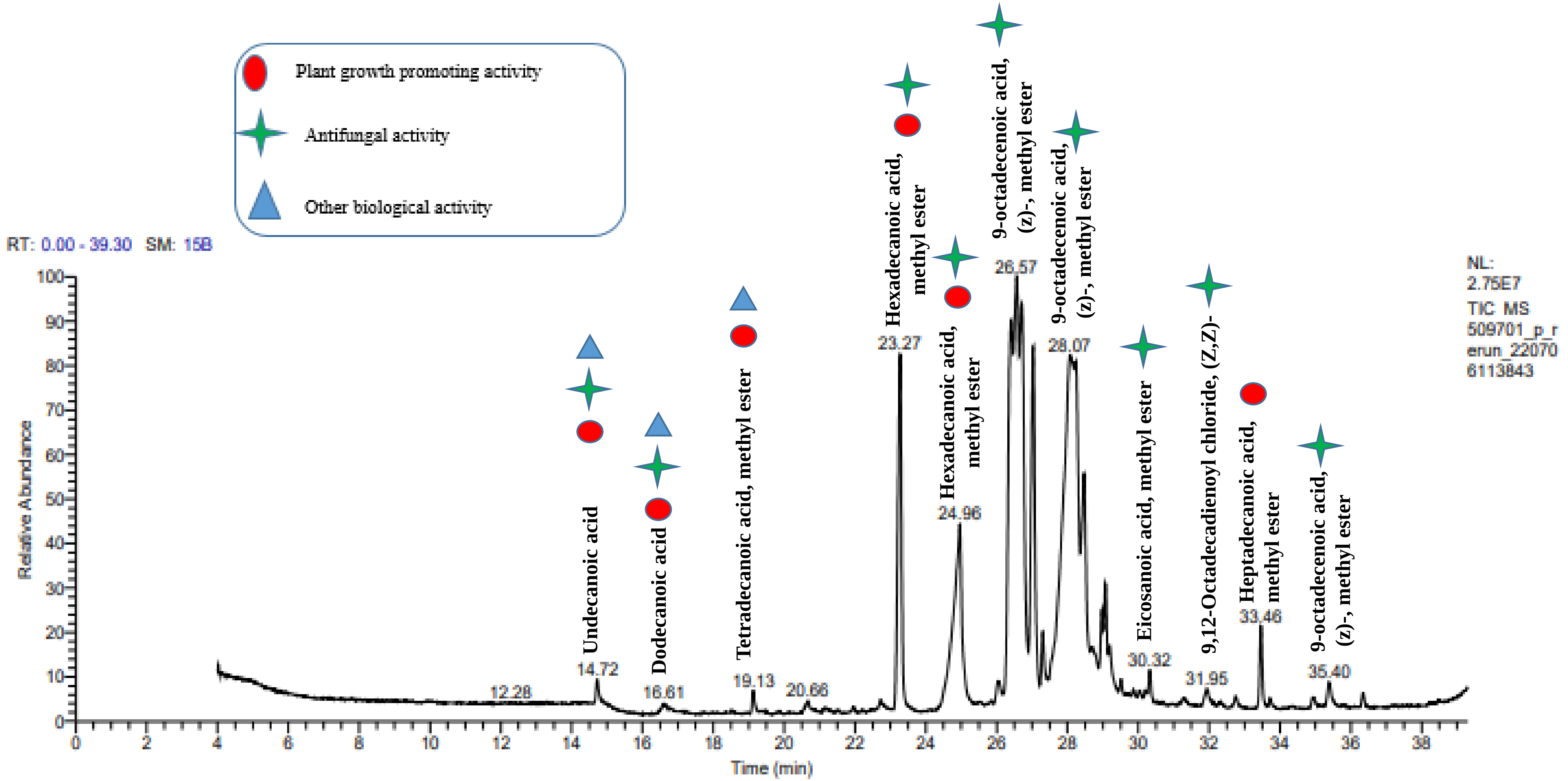

Fig. S9: Depicts the GC-mass chart for VOCs emitted by the HNA3 strain using headspace-solid phase microextraction. The compound names are indicated above each peak, while different shapes (circle, triangle, and star) represent the known biological functions of each compound.

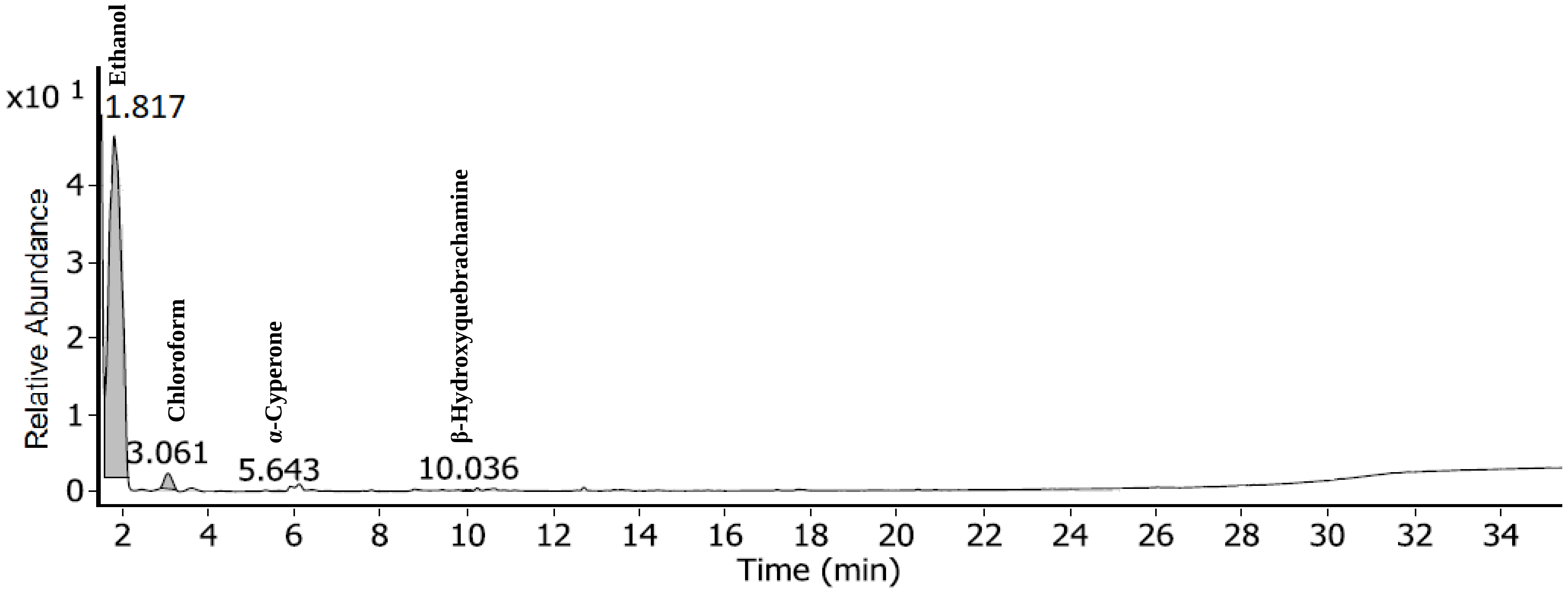

Fig. S10: Illustrates the GC-mass chart of emitted VOCs collected from the headspace of TSA media without bacteria (control). The compound names are indicated above each peak.

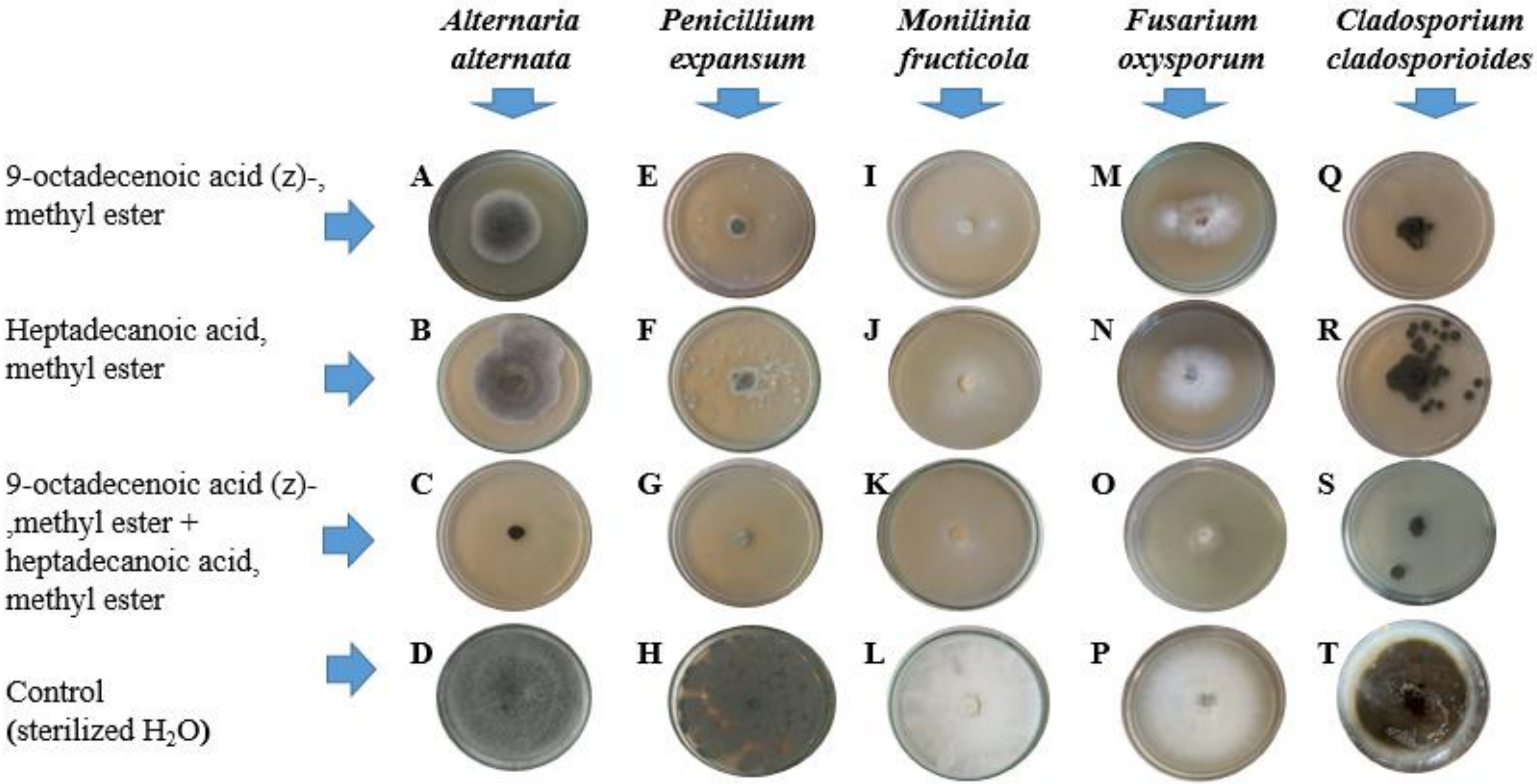

Fig. S11: Demonstrates the antifungal effect of pure VOCs emitted from *Bacillus velezensis* HNA3 against 5 postharvest pathogenic fungi. (A, E, I, M, and Q) Show that 9-octadecenoic acid (z)-, methyl ester inhibited the growth of all pathogenic fungi. (B, F, J, N, and R) Indicate that heptadecanoic acid, methyl ester showed moderate inhibition activity against pathogenic fungi. (C, G, K, O, and S) Depict the results of the VOCs mixture, consisting of 9-octadecenoic acid (z)-, methyl ester and heptadecanoic acid, methyl ester, which eradicated the growth of fungal mycelia. (D, H, L, P, and T) Represent the control group where sterilized H2O was used instead of volatile compounds.
